# Assessing methodological differences in estimating exposure to intimate-partner violence against women in Brazil: a multi-source observational analysis

**DOI:** 10.1016/j.lana.2026.101632

**Published:** 2026-09-12

**Authors:** Caroline Stein, Nádia Machado de Vasconcelos, Carolina VN. Coll, Joseph Murray, Franciele Marabotti Costa Leite, Bruna Venturin, Luisa Sorio Flor, Simon I. Hay, Molly Herbert, Aisha Twalibu, Emmanuela Gakidou, Deborah Carvalho Malta

**Affiliations:** a Institute for Health Metrics and Evaluation, University of Washington, Seattle, WA, USA; b Department of Health Metrics Sciences, School of Medicine, University of Washington, Seattle, WA, USA; c Federal University of Minas Gerais, Belo Horizonte, Minas Gerais, Brazil; d Department of Epidemiology, Federal University of Pelotas, Pelotas, Brazil; e Human Development and Violence Research Center, Federal University of Pelotas, Pelotas, Brazil; f Nursing Department, Federal University of Espírito Santo, Brazil; g Postgraduate Program in Epidemiology, Federal University of Pelotas, Pelotas, Brazil

**Keywords:** Intimate-partner violence, Violence against women, Epidemiology

## Abstract

**Background:**

Intimate partner violence (IPV) is a global public health issue. However, prevalence estimates vary depending on the data source and methodology used. This study aimed to assess methodological differences in estimating the prevalence and age distribution of physical and sexual IPV across multiple Brazilian data sources.

**Methods:**

We assessed and analyzed seven Brazilian data sources: five surveys and two administrative datasets. Surveys used either the World Health Organization (WHO) instrument on violence against women and adaptations or an adapted version of the Revised Conflict Tactics Scales. The analysis included only women aged 16 years or older, and race was not examined. Recall periods varied from 12 to 24 months. We estimated prevalence and age distribution of physical and sexual IPV with 95% confidence intervals (CIs).

**Findings:**

The seven sources represented national, regional, and local populations. Methodological differences, including questionnaires, interview settings, data collection methods, and recall periods, may explain variation across data sources. Among national-level estimates, physical IPV prevalence ranged from 0.4% (0.3–0.7) to 13.0% (8.3–17.8) across data sources and age strata, with all sources consistently showing higher prevalence and proportions among younger women. For sexual IPV, no age group pattern emerged in prevalence or proportions.

**Interpretation:**

This study revealed substantial heterogeneity in estimates of IPV prevalence and age distribution. Without a gold standard for IPV measurement, these findings underscore the need to interpret available estimates in light of the methodological context in which they were generated.

**Funding:**

The Gates Foundation.

## Resumo

**Contexto:**

A violência por parceiro íntimo (VPI) é um problema de saúde pública global. Entretanto, as estimativas de prevalência variam dependendo da fonte de dados e da metodologia utilizada. Este estudo objetivou avaliar diferenças metodológicas na estimativa da prevalência e da distribuição etária da VPI física e sexual em múltiplas fontes de dados brasileiras.

**Métodos:**

Avaliamos e analisamos sete fontes de dados brasileiras: cinco inquéritos e dois sistemas administrativos. Os inquéritos utilizaram o instrumento da Organização Mundial da Saúde (OMS) sobre violência contra a mulher e suas adaptações ou uma versão adaptada da Revised Conflict Tactics Scales. A análise incluiu apenas mulheres com 16 anos ou mais e a variável raça não foi analisada. Os períodos recordatórios variaram de 12 a 24 meses. Estimamos a prevalência e a distribuição etária da VPI física e sexual com seus respectivos intervalos de confiança de 95% (IC 95%).

**Achados:**

As sete fontes de dados representaram a população a nível nacional, regional e local. Diferenças metodológicas, incluindo questionários, contexto das entrevistas, métodos de coleta de dados e períodos recordatórios, podem explicar a variação entre as fontes de dados. A nível nacional, a prevalência de VPI física variou de 0.4% (0.3–0.7) a 13.0% (8.3–17.8) entre as fontes e as faixas etárias, com todas elas mostrando consistentemente maior prevalência e maior proporção entre as mulheres mais jovens. Para violência sexual, não houve um padrão etário, nem para prevalência, nem para proporção de casos.

**Interpretação:**

Este estudo revelou uma heterogeneidade substancial nas estimativas da prevalência e da distribuição etária da VPI. Na ausência de um padrão-ouro para mensuração da VPI, esses achados mostram a necessidade de interpretar os dados disponíveis à luz do contexto metodológico em que eles foram gerados.

**Financiamento:**

Fundação Gates.

**Research in context**

**Evidence before this study**

There is no systematic review study available in the literature on the methodological assessment of the exposure estimation of intimate partner violence (IPV) in Brazil. Despite this, Brazil is recognized for producing a substantial body of research on violence against women. On June 23, 2026, we conducted a search in PubMed without restrictions on language, publication date, or other filters. The search strategy “intimate partner violence” AND “Brazil” identified 456 studies. Adding AND “systematic review” to the search strategy narrowed the results to 34 studies. Among these, most focused on IPV in relation to other outcomes (e.g., cervical cancer, cash transfer programs, orofacial injuries, and HIV) or specific populations (elderly, LGBTQ+ and adolescents). Four studies reported supranational prevalence estimates (e.g., the Americas, the Caribbean, and Ibero-America), while three relied on their own methodological approaches, such as the Global Burden of Disease (GBD) framework. None of the identified studies included all available data sources from Brazil reporting IPV. Previous research indicates that estimates of IPV prevalence can vary greatly depending on the type of data used and the methodological approach. Understanding the true extent of IPV exposure remains a challenge, highlighting the need for additional research in this field.

**Added value of this study**

To date, Brazilian studies estimating the exposure to IPV have been published individually. Our study assesses multiple data sources used to estimate IPV against women in Brazil and identifies substantial heterogeneity in how IPV is measured. Differences in questionnaires, IPV definitions, study populations, and other methodological choices are reflected in the estimates generated by each data source. Our findings indicate that these methodological differences may shape the prevalence and age distribution of IPV cases captured by different data sources. By assessing these differences across multiple data sources within a single country, this study contributes to a more nuanced understanding of the evidence available on IPV and the challenges involved in interpreting its estimated magnitude.

**Implications of all the available evidence**

Based on methodological differences, we found variability results in the prevalence of physical and sexual IPV and age distribution of cases. It is evident that understanding the nuances of different data collection approaches is essential for improving IPV assessments and refining strategies to strengthen public policies globally. Brazil’s vast geographic, demographic, and administrative diversity makes it a particularly relevant setting to examine IPV. This heterogeneity mirrors the complexity found in many middle-income countries, enhancing the generalizability of findings. Key factors influencing measurement include differences in questionnaires, sample sizes, research scope (national, regional, or local), and violence notification systems. There is an urgent need to enhance how violence against women is measured, which includes adopting best practices in data collection, standardizing inquiry and survey techniques, and establishing clear guidelines for future initiatives. Such advancements are critical to addressing the issue of underreported IPV and obtaining more accurate estimates of the number of women affected. Using research methodologies that maximize IPV case identification can produce more reliable data that reflect the true scope of violence against women. Ultimately, these improvements are expected to drive substantial policy investments in victim protection, as well as the development of robust detection and prevention strategies for this pervasive health risk factor affecting countless women worldwide.

## Introduction

Intimate partner violence (IPV) is a global public health problem, affecting women of all ages and across socioeconomic conditions.^1^^,^^2^ In 2021, the Global Burden of Diseases, Injuries, and Risk Factors Study (GBD) estimated that 22.0% (uncertainty interval [UI] 14.5–24.2) of women aged 15 or older worldwide had experienced physical and/or sexual IPV in their lifetime.^3^ The prevalence was 20.1% (UI 13.8–22.8%) in Latin America and the Caribbean, and 17.3% (UI 10.6–22.0) in Brazil.^2^ Despite being declared a public health priority by the World Health Organization (WHO) in 1996, IPV remains challenging to measure accurately.^4^

These challenges stem from multiple factors that affect measurement and reporting of violence results, such as stigma, safety, confidentiality, and privacy concerns.^5^^,^^6^ Additionally, variations in data collection methods and interviewing techniques (including interview setting and whether face-to-face, self-administered, or anonymous questionnaires were used) contribute to differences across studies.^7^, ^8^, ^9^, ^10^ Inconsistencies also arise from differences in recall period (lifetime, last two years, or past year), definitions of violence (domestic violence, IPV, sexual, physical, psychological violence), types of perpetrators (anyone, partner, family member, unknown), and inclusion of other forms of violence (e.g., economic, witness, female genital mutilation, cyberviolence).^11^^,^^12^ These limitations highlight the persistent challenges in approaching gender-based violence. Efforts to improve violence measurement should prioritize standardized survey methodologies and instruments,^13^^,^^14^ including explicit recall periods and the avoidance of restrictive filter questions.^15^ Another point is that repeated measurement of IPV in longitudinal surveys and clinical settings may provide additional opportunities to identify women exposed to violence.^16^^,^^17^ Incorporating data from multiple sources is also important for improving the measurement of violence against women. These sources include Civil Police records, which play a fundamental role in documenting and responding to IPV cases.^10^^,^^18^

Currently, there is no gold standard for measuring violence against women, highlighting the need for more reliable and valid methods to assess IPV. Brazil, the fifth-largest country by area and the seventh most populous, has various data sources that collect information on IPV. These sources differ in target populations, data collection methods, and analysis periods. This study aimed to assess how methodological variability across data sources influences estimates of physical and sexual IPV and to systematically characterize differences in prevalence estimates and age distribution of IPV cases across multiple Brazilian data sources. By doing so, we sought to identify potential reporting biases to inform future studies and effective public health strategies.

## Methods

### Overview

This is an epidemiological study using seven different data sources from Brazil. According to the 2022 census, Brazil has 203,080,756 inhabitants (51.5% are women - 104,548,325).^19^ In this study, we included data on physical (defined as the use of physical force by a current or former intimate partner) and sexual (referred to any non-consensual sexual act or attempt within an intimate relationship) IPV against women that occurred in the last 12 or 24 months, with data with representativeness from Brazil (National level), Espírito Santo (State level), Vitória (capital level), and Pelotas (municipal level). This study complies with the Guidelines for Accurate and Transparent Health Estimates Reporting (GATHER) and STROBE checklist for cross-sectional studies (Supplementary information Tables S1 and S2).^20^^,^^21^ This study included estimates referring only to women and were derived from the sex-related available data. We used the gender-specific term women in the presentation and interpretation of the findings to ensure more inclusive terminology. Race or ethnicity data were not included in this study.

### Data sources

We identified publicly available and governmental datasets, supported by a review of academic literature and official reports on IPV measurement in Brazil. Inclusion criteria focused on data collected within the last approximately 20 years to ensure contemporary relevance, and on datasets that included variables on physical and sexual IPV, as well as adequate demographic information, particularly age groups. We included studies that reported recent exposure to IPV, but excluded those reporting lifetime exposure, to better reflect current patterns of violence. The seven selected datasets were chosen for their national or regional coverage, their capture of Brazil’s geographic and demographic diversity, and their methodological variety—including household surveys, health surveillance, and violence notification systems—enabling analysis of IPV prevalence estimates.

The main characteristics of survey and administrative data sources are presented in Table 1. Below, we provide a brief overview of each data source included in this analysis; additional details are provided in Section 3 of the Supplementary Information.

**Table 1:** Summary of key methodological considerations for each survey and administrative data source included in the study, categorized by geographic level (national, state, capital, or municipal).

Data source	Year of event	Recall Type	Age	Sample	Cases	Measured Indicator	Methodological considerations
Survey – National level (Brazil)							
National Health Survey (PNS)	2019	Last 12 months	18 years old or more	Interviews: in 90,846 households, 46,869 women interviewed Expanded sample: 84,618,612 women. Probabilistic sample by clusters in three selection stages: census sectors or set of census sectors (primary units); households (secondary units); and residents (tertiary units).	Physical IPV: 1000 cases Sexual IPV: 244 cases	Physical and sexual IPV that occurred in the last 12 months	Population-based survey Household face-to-face interview Adapted WHO instrument to initially investigate violence without specifying the perpetrator. Participants who reported experiencing violence in the past 12 months were then asked to report the person responsible for the “most severe episode.” Sample-weight variable used: V00291
Brazilian National Alcohol and Drugs Survey (LENAD)	2006	Last 12 months	16 years old or more	Interviewed: 811 women Expanded sample: 37,315,317 women	Physical IPV: 77 cases	Physical IPV that occurred in the last 12 months	Household face-to-face interview Adapted WHO instrument Only married women Current partner
	2012		16 years old or more	Interviewed: 1199 women Expanded sample: 44,279,465 women	Physical IPV: 78 cases	Physical PV that occurred in the last 12 months	LENAD 2006 Sample-weight variable used: weight_4 LENAD 2012 Sample weight variable used: final_weight
Data Senado Research (DSR)	2019	Last 12 months	16 years old or more	Interviewed: phone survey: 2400 women Expanded sample: 86,658,731 women	Physical IPV: 184 cases	Physical IPV in the last 12 months	Landline or cell phone interview Adapted WHO instrument Sample-weight variable used: W2
Administrative data – National level (Brazil)							
Brazil’s Information System for Notifiable Diseases (SINAN)	2019	Cases reported in 2019	16 years old or more	–	Physical IPV: 62,265 cases Sexual IPV: 3550 cases	Case of violence reported by a health professional	Health system notification Mandatory notification of violence by health professional Underreporting is estimated at around 90% when compared to surveys
Administrative data – State level (Espírito Santo)							
Brazil’s Information System for Notifiable Diseases (SINAN)	2019	Cases reported in 2019	18 years old or more	–	Physical IPV: 1311 cases Sexual IPV: 80 cases	Case of violence reported by a health professional	Health system notification Mandatory notification by health professional
Civil Police	2019	Cases reported in 2019	18 years old or more	–	Physical domestic violence: 1106 cases Sexual violence: 449 cases (all cases)	Case of violence reported by a civil police officer	Incidents are the criminal types registered in the Police System and are based on the penal code The guideline when registering an incident is to choose the most severe criminal type Incident Maria da Penha Law: most of them are Intimate partner violence cases but could include other domestic violence from other perpetrators
Survey – Capital level (Vitória)							
Vitória study	2020–2021	Last 24 months	18 years old or more	Interviewed: 1086 women	Physical IPV: 98 cases Sexual IPV: 71 cases	Physical and sexual IPV that occurred in the last 24 months	Population-based survey Household Face-to-face interview - Privacy guaranteed WHO instrument validated for use in Brazil Data collection 2022 referring to last 24 months violence during COVID-19 pandemic
Administrative data – Capital level (Vitória)							
Brazil’s Information System for Notifiable Diseases (SINAN)	2020–2021	Cases reported in 2020–2021	18 years old or more	–	Physical IPV: 46 cases	Case of violence reported by a health professional	Health system notification Mandatory notification by health professional
Civil Police	2020–2021	Cases reported in 2020–2021	18 years old or more	–	Physical domestic violence: 290 cases Sexual violence: 153 cases (all cases)	Case of violence reported by a civil police officer	Incidents are the criminal types registered in the Police System and are based on the penal code The guideline when registering an incident is to choose the most severe criminal type Incident Maria da Penha Law: most of them are Intimate partner violence cases but could include other domestic violence from other perpetrators
Survey – Municipal level (Pelotas)							
Pelotas study	2019	Last 12 months	18 years old or more	Interviewed: 4266 women	Physical IPV: 281 cases Sexual IPV: 58 cases	Physical and sexual IPV that occurred in the last 12 months	Population-based survey Assessment in university setting Face-to-face interview WHO Instrument All participants are mothers with young children
Administrative data – Municipal level (Pelotas)							
Brazil’s Information System for Notifiable Diseases (SINAN)	2019	Cases reported in 2019	18 years old or more	–	Physical IPV: 29 cases	Case of violence reported by a health professional	Health system notification Mandatory notification by health professional

### Survey data sources

The 2019 Brazil National Survey of Health (PNS - Pesquisa Nacional de Saúde, in Portuguese) is a household-based, population-based study that employed a complex multistage sampling design, representative of Brazil, its subunits, and the Federal District.^22^ The survey included a Violence Module, applied to individuals aged 18 and older, which utilized an adapted version of the World Health Organization (WHO) instrument on violence against women.^23^ This module assessed physical and sexual IPV, employing a modified version of the WHO instrument to first assess experiences of violence without identifying the perpetrator. Respondents who reported incidents within the past 12 months were subsequently asked to specify who was responsible for the “most severe episode” (Supplementary information, Section 3). More details about this module can be found in a previous study.^24^

Data Senado Research (DSR) is a telephone survey representative of Brazil that utilized an adapted version of the World Health Organization (WHO) instrument on violence against women. A total of 2400 interviews were conducted via phone.^25^^,^^26^ Only data on physical IPV among women 16 and older were included, as the 2019 DSR study did not collect information on sexual IPV within the last 12 months (Supplementary information, Section 3). The data were accessed through the Data Senado website.^25^

The Brazilian National Alcohol and Drugs Survey^27^^,^^28^ (LENAD – Levantamento Nacional de Alcool e Drogas, in Portuguese) is a household survey that utilized an adapted version of the Revised Conflict Tactics Scales to measure physical IPV occurring in the last 12 months among women aged 16 and older, married or living with a partner, representative of Brazil. Data were collected during two periods: LENAD I (November 2005–April 2006) and LENAD II (2012).^29^ Analyses for sexual IPV were not performed due to the low number of cases identified (Supplementary information, Section 3).

A population-based study conducted in Vitória,^30^ the capital of Espírito Santo, was carried out between January and May 2022 to investigate violence against women during the COVID-19 pandemic. The study utilized the World Health Organization (WHO) instrument on violence against women.^23^^,^^30^ The analysis focused on physical and sexual IPV perpetrated by a current or former intimate partner within the past 24 months against women aged 18 and older, representative of Vitória (Supplementary information, Section 3).

The 2015 Pelotas Birth Cohort is a longitudinal study designed to monitor the health of all children born in Pelotas between January 1 and December 31, 2015.^31^^,^^32^ Physical and sexual IPV was measured using the World Health Organization (WHO) instrument on violence against women.^23^^,^^33^ For the current study, data were collected during the 4-year follow-up in 2019 using REDCap^34^ as the instrument for data collection, focusing on IPV that occurred in the last 12 months among all mothers who gave birth in Pelotas in 2015 (Supplementary information, Section 3).

### Administrative data sources

Brazil’s Information System for Notifiable Diseases (SINAN - Sistema de Informação de Agravos de Notificação, in Portuguese) is a national information system for reporting and investigating cases of diseases and conditions that are notifiable and subject to mandatory notification.^35^ In the current analysis, data on physical IPV cases against women aged 16 and older for National (Brazil), subnational (Espírito Santo), capital (Vitória), and municipality (Pelotas) geographic levels were included. For sexual IPV cases, only National (Brazil) and subnational (Espírito Santo) data were analyzed, because in Vitória (capital) and Pelotas (municipality) a low number of cases was identified, which precluded their inclusion in the analyses. SINAN data were not used to estimate prevalence at any level, as the system captures reported cases of violence rather than the overall occurrence within the population. Instead, SINAN data were utilized to analyze the age distribution of IPV cases by type (Supplementary information, Section 3).

The Civil Police System from the state of Espírito Santo is part of the State Secretariat for Public Security and Social Defense.^36^ In the current analysis, only data on physical IPV and sexual violence perpetrated by anyone against women 18 years old or above, from the State of Espírito Santo (2019) and Vitória (2020–2021) were included. Civil Police data were not used in prevalence estimates at any level, as they refer to reported cases of violence rather than the overall occurrence of violence in the population. Therefore, Civil Police data were included only in the age distribution of cases analysis by type of IPV (Supplementary information, Section 3).

### Variables

The study used the following variables from each data source: year of event, participant age (categorized as 16–29, 30–39, 40–49, 50–59, and 60–99), and type of violence (physical and sexual IPV). IPV was classified based on a “yes” or “no” response. The IPV variable encompassed questions related to physical and sexual IPV, tailored to each data source. For instance, if a data source included three questions about physical IPV and a participant answered “yes” to at least one, the case was counted as a prevalent instance of physical IPV. Details on all questions used to identify physical and sexual IPV cases in this study are available in Supplementary Tables S3 and S4.

### Statistical analysis

First, the data sources were identified based on the inclusion and exclusion criteria. Then, an evaluation was conducted to map the main characteristics and variables of the data sources, including target audience, definition of violence, interview format, and data collection instrument. Subsequently, prevalence and age distribution of IPV cases were estimated with 95% confidence intervals (CIs) across data sources by type of IPV (physical, sexual) and age groups (16–29, 30–39, 40–49, 50–59, 60–99).

Prevalence estimates were calculated separately for each survey data source and age group. For survey-based datasets with available sampling weights, weighted estimates were calculated using the corresponding sample-weight variables. For the 2006 LENAD, 2012 LENAD, and 2019 DSR datasets, analyses were conducted in R using survey-weighted procedures; for the 2019 PNS, analyses were conducted in Stata accounting for the complex survey design, including sampling weights, primary sampling units, and strata. Vitória estimates were reported from crude frequencies in a multi-stage population-based sample, while Pelotas estimates were calculated as unweighted age-specific prevalences. The 95% confidence intervals for prevalence estimates were calculated using methods appropriate to each data source, accounting for sampling weights and survey design when available. Additional details on the data-source-specific procedures are provided in the Supplementary information, Section 3.

The age distribution of IPV cases was estimated as the proportion of all physical and sexual IPV cases occurring within each age group. For survey-based datasets with sampling weights, the distribution was calculated using weighted cases, whereas for administrative data sources, including SINAN and the Civil Police records, crude case counts were used. The 95% confidence intervals for the age distribution of cases were calculated using approaches consistent with the structure of each data source, incorporating survey design features when available and using crude proportions for administrative data. The age distribution of cases was not estimated for the Pelotas Birth Cohort because the sample consisted almost entirely of young women, and consequently nearly all cases occurred within the same age group. Missing data were handled using available-case analysis. Further methodological details are presented in the Supplementary information, Section 3. Analyses were conducted using Stata (version 13.1) and R (version 4.6.0).

### Ethical aspects

This study used secondary data from public databases and, therefore, did not require approval from the Research Ethics Committee, according to Resolution no. 466/2012 of the Brazilian National Health Council.

### Role of the funding source

The founder of this study had no role in study design, data collection, data analysis, data interpretation, or the writing of the report. The lead and senior authors had full access to the study data and final responsibility for the decision to submit it for publication.

## Results

### Overview

We analyzed seven data sources from Brazil, covering different geographic levels of representativeness, ranging from national to municipal levels. The sources included in-person interviews conducted at women’s homes or at universities, telephone interviews, health-sector mandatory reporting data, and police reports. One survey (PNS) focused on women aged 18 and older, while the others included those aged 16 and older. One source interviewed mothers exclusively, and another targeted married woman or those living with partners. Most surveys examined IPV within the past 12 months, except for the Vitória survey, which investigated IPV during the COVID-19 pandemic (2020–2021) over a 24-month period (Table 1).

The sources varied in their approaches to measuring IPV. We included all questions from each data source used in this study in Supplementary Tables S3 and S4. The LENAD 2006 and 2012 surveys used an adapted version of the Revised Conflict Tactics Scales, while studies in Vitória and Pelotas applied the WHO instrument on violence against women without modification. PNS and DSR also used the WHO instrument but with different adaptations. Consequently, all surveys included questions about specific acts of IPV, although the number and types of questions differed. For example, DSR 2019 included two questions on physical IPV, whereas LENAD 2006 and 2012 included eight. For sexual IPV, surveys ranged from one to three questions. The PNS uniquely used an adapted WHO instrument to initially investigate violence without specifying the perpetrator. Participants who reported experiencing violence in the past 12 months were then asked to identify the perpetrator of the “most severe episode”.

Health sector notification data (SINAN) defined physical and sexual IPV according to WHO classifications, with detailed descriptions of acts constituting these forms of violence. Civil Police data on physical violence relied on domestic violence filed under the Maria da Penha Law, Brazil’s first federal law addressing violence against women.^37^ Due to the lower number of registered sexual violence cases in Civil Police data, all such cases were included in the analysis, including those classified as domestic violence under the Maria da Penha Law.

### Surveys data sources

The PNS is a household survey conducted among individuals aged 18 years or older, including 46,869 women. The LENAD is a household survey targeting individuals aged 14 years or older who were married or living with a partner. For this study, we included interviews with women aged 16 years or older, resulting in 811 women interviewed in 2006 and 1119 in 2012. The Data Senado Research is conducted by telephone among women aged 16 years or older and included 2400 interviews in 2019. At the capital level, the Vitoria study is a household survey among women aged 18 years or older which included 1086 women, whereas at the municipal level, the Pelotas study surveyed mothers aged 18 years or older at a university, including 4266 women (Table 1).

**Fig. 1:**
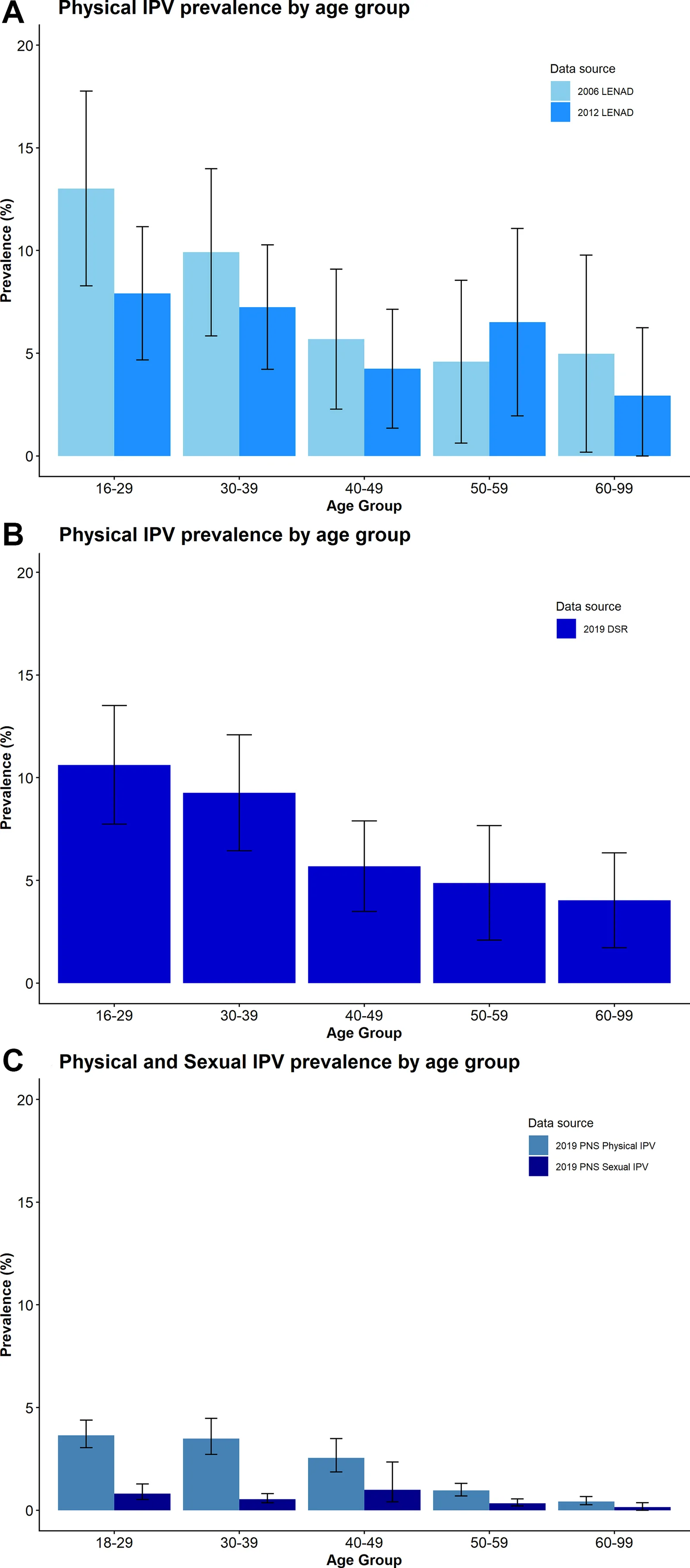
Prevalence of physical and sexual intimate partner violence (IPV) with 95% confidence intervals (CIs) across survey data sources and age groups at the national level, Brazil. (A) Physical IPV prevalence by age group, 2006 LENAD (National Alcohol and Drugs Survey) and 2012 LENAD (National Alcohol and Drugs Survey), (B) Physical IPV prevalence by age group, 2019 DSR (Data Senado Research), and (C) Physical and sexual IPV prevalence by age group, 2019 PNS (National Survey of Health).

Regarding physical IPV, although overall prevalence varied across data sources, a consistent age-group pattern emerged, with higher prevalence among younger women. Nationally, the highest prevalence estimates were observed among women aged 16/18–29, ranging from 13.0% (8.3–17.8) in LENAD 2006 (Fig. 1A, Table 2) to 3.6% (3.0–4.4) in PNS 2019 (Fig. 1C, Table 2), representing more than a threefold difference. Across all age groups, PNS 2019 consistently reported the lowest prevalence of physical IPV (Fig. 1C, Table 2). At capital and municipal level studies, the patterns in prevalence of physical IPV persisted, with prevalence among women aged 18–29 ranging from 13.4% (9.3–18.8) in the 2020–2021 Vitória study (Supplementary Fig. S1 and Table S5) to 7.5% (6.3–8.7) in Pelotas (Supplementary Fig. S2 and Table S5).

**Table 2:** Prevalence of physical and sexual intimate partner violence (IPV) with 95% confidence intervals (CI) across survey data sources and age groups at the national level, Brazil.

Data source	Age group	Study sample	Cases	Prevalence (95% CI)
Physical IPV				
2006 LENAD	16–29	210	31	13.0 (8.3–17.8)
	30–39	246	25	9.9 (5.8–14.0)
	40–49	166	12	5.7 (2.3–9.1)
	50–59	104	5	4.6 (0.6–8.6)
	60–99	85	4	5.0 (0.2–9.8)
2012 LENAD	16–29	359	33	7.9 (4.7–11.2)
	30–39	306	22	7.2 (4.2–10.3)
	40–49	227	9	4.2 (1.4–7.1)
	50–59	171	11	6.5 (1.9–11.1)
	60–99	136	3	2.9 (0.0–6.2)
2019 DSR	16–29	588	64	10.6 (7.7–13.5)
	30–39	592	55	9.3 (6.4–12.1)
	40–49	488	35	5.7 (3.5–7.9)
	50–59	370	16	4.9 (2.1–7.7)
	60–99	362	14	4.0 (1.7–6.3)
2019 PNS	18–29	8073	327	3.6 (3.0–4.4)
	30–39	9354	322	3.5 (2.7–4.5)
	40–49	8644	197	2.5 (1.9–3.5)
	50–59	8263	107	1.0 (0.7–1.3)
	60–99	12,535	47	0.4 (0.3–0.7)
Sexual IPV				
2019 PNS	18–29	8073	64	0.8 (0.5–1.3)
	30–39	9354	70	0.5 (0.4–0.8)
	40–49	8644	57	1.0 (0.4–2.4)
	50–59	8263	36	0.3 (0.2–0.5)
	60–99	12,535	17	0.1 (0.0–0.4)

2006 LENAD (National Alcohol and Drugs Survey), 2012 LENAD (National Alcohol and Drugs Survey), 2019 PNS (National Survey of Health), and 2019 DSR (Data Senado Research).

**Fig. 2:**
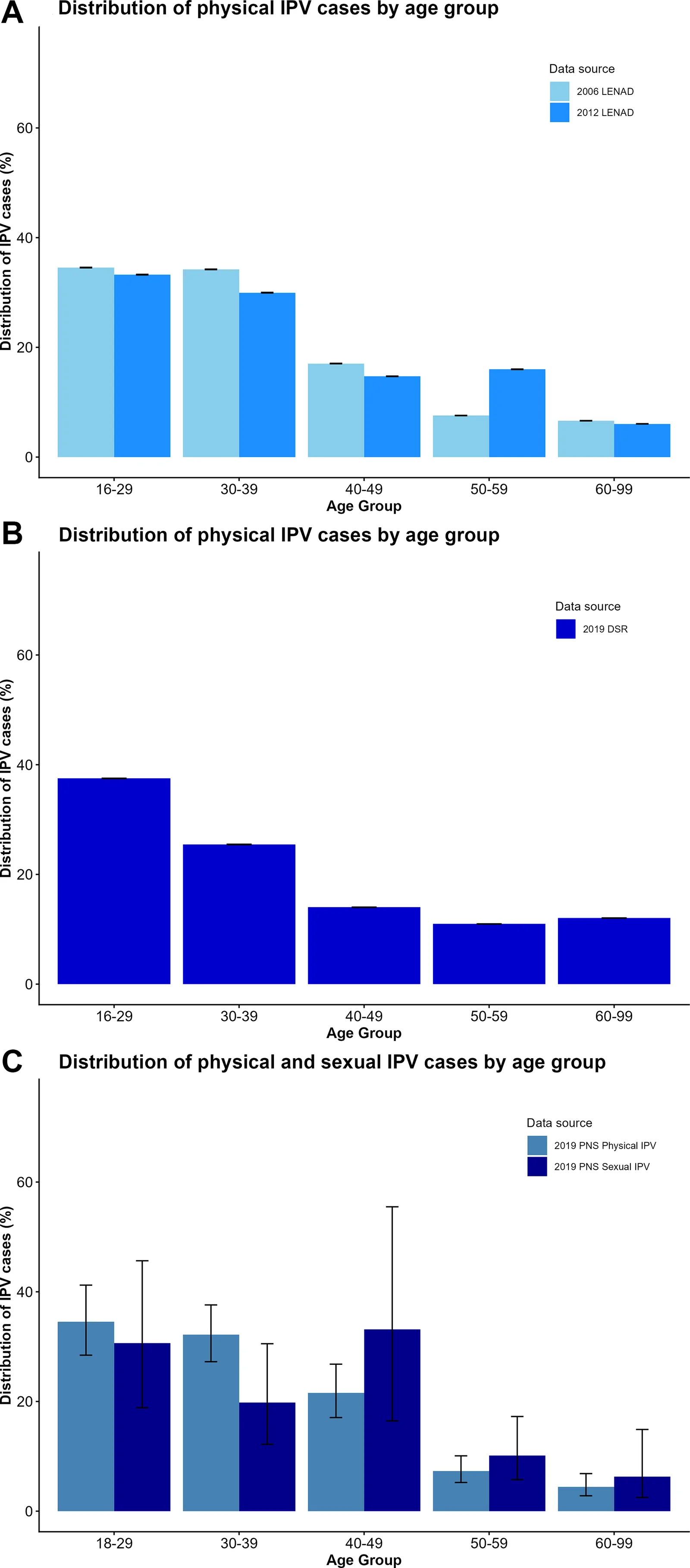
Age distribution of physical and sexual intimate partner violence (IPV) cases with 95% confidence intervals (CIs) across survey data and age groups at the national level, Brazil. (A) Distribution of physical IPV cases by age group, 2006 LENAD (National Alcohol and Drugs Survey) and 2012 LENAD (National Alcohol and Drugs Survey), (B) Distribution of physical IPV cases by age group, 2019 DSR (Data Senado Research), and (C) Distribution of physical and sexual IPV cases by age group, 2019 PNS (National Survey of Health).

Regarding the age distribution of physical IPV, at the national level, all data sources showed a greater proportion of cases among women aged 18–29 and 30–39 (Fig. 2, Supplementary Table S6). For example, 2019 PNS found the highest proportions among women aged 18–29 (34.5% [28.4–41.2]) and 30–39 (32.2% [27.3–37.6]), followed by a progressive decline in older age groups, reaching 4.4% (2.8–6.8) among women aged 60 and older (Fig. 2, Supplementary Table S6). At the capital level, the 2020–2021 Vitória study showed a more even distribution of physical IPV cases across age groups (Supplementary Fig. S3, Supplementary Table S7).

For sexual IPV, at the national level, prevalence in PNS 2019 peaked at 1.0% (0.4–2.4) among women aged 40–49 years (Fig. 1C, Table 2). At the capital level, the 2020–2021 Vitória study reported higher prevalence estimates, ranging from 3.4% (1.8–6.5) among women aged 60 and older to 8.9% (5.7–13.7) in the 18–29 age group (Supplementary Fig. S1, Supplementary Table S5). At the municipal level, the 2019 Pelotas study recorded the highest prevalence at 1.7% (0.5–2.8) in the 40–49 age group (Supplementary Fig. S2, Supplementary Table S5).

The age distribution of sexual IPV varied across surveys and geographic levels. Nationally, 2019 PNS found the highest proportions among women aged 40–49 (33.1% [16.5–55.5]) and 18–29 (30.6% [18.9–45.6]), with lower proportions observed in older groups, including 6.3% (2.5–14.9) among women aged 60 and older (Fig. 2C, Supplementary Table S6). At the capital level, the 2020–2021 Vitória study showed no clear age pattern for sexual IPV, reporting identical proportions of 25.4% (16.6–36.7) among women aged 18–29 and 40–49, and 12.7% (6.7–22.6) among women aged 60 and older (Supplementary Fig. S3, Supplementary Table S7).

### Administrative data sources

For administrative data, SINAN 2019 provided information at national, state, capital, and municipal levels. At the national (Brazil) level, 62,265 cases of physical IPV and 3550 cases of sexual IPV were included; at the state level (Espírito Santo), 1311 cases of physical IPV and 80 cases of sexual IPV; at the capital level (Vitória), 46 cases of physical IPV; and at the municipal level (Pelotas), 29 cases of physical IPV. Civil Police data were available at the state (Espírito Santo) and capital (Vitória) levels, with 1106 cases of physical domestic violence and 449 cases of sexual violence reported at the state level, and 290 cases of physical domestic violence and 153 cases of sexual violence reported in the capital (Table 1).

**Fig. 3:**
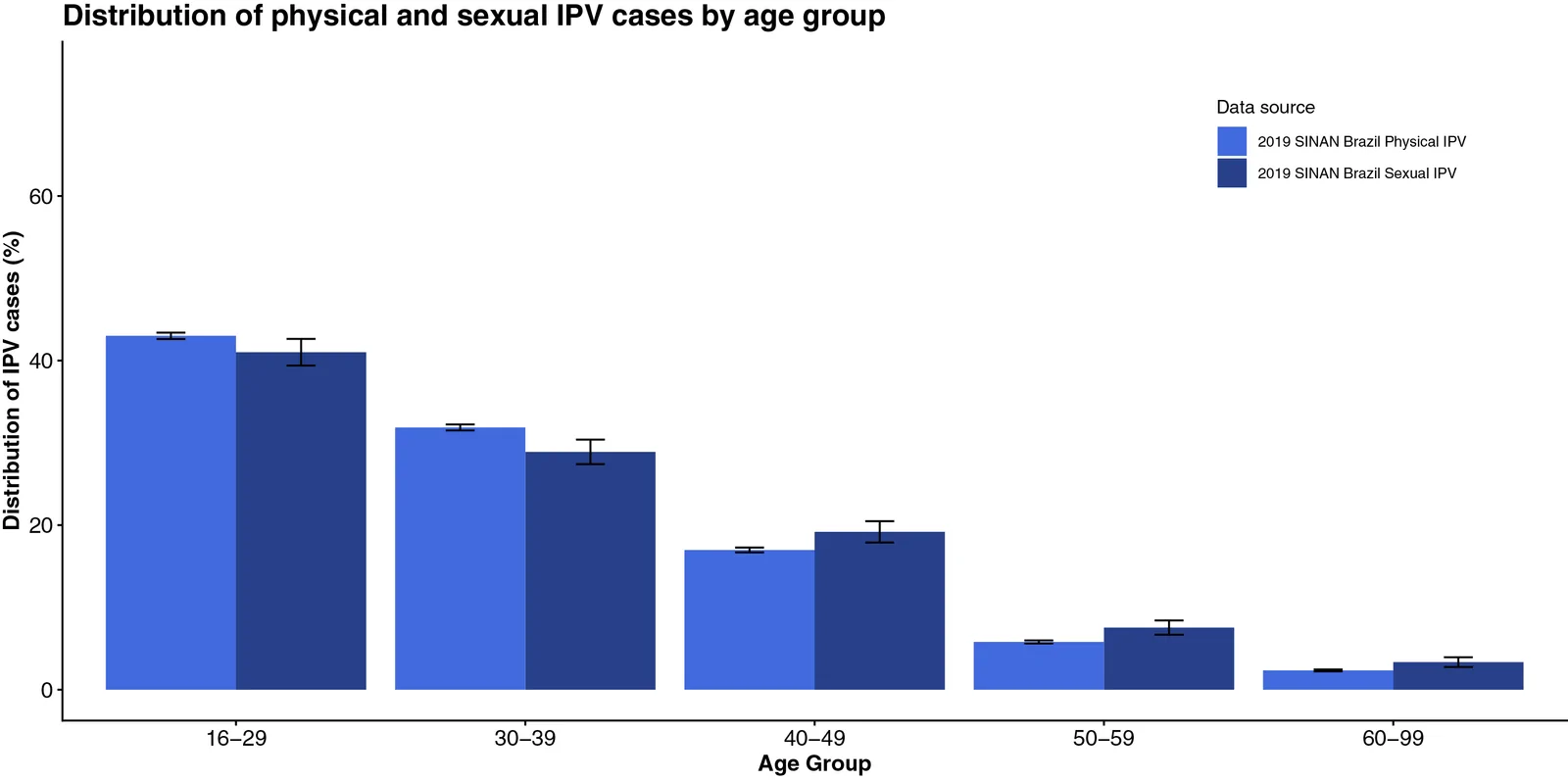
Distribution of physical and sexual intimate partner violence (IPV) cases with 95% confidence intervals (CIs) for the 2019 Information System for Notifiable Diseases (SINAN), at the national level, Brazil.

For administrative data sources, we analyzed the age distribution of reported physical and sexual IPV cases. For physical IPV, SINAN 2019 showed a higher proportion of reports among women aged 16–29 (43.0 [42.6–43.4]) at the national level, followed by women aged 30–39 (31.9 [31.5–32.2]) (Fig. 3, Supplementary Table S8). At the state level (Espírito Santo), both the 2019 SINAN (Supplementary Fig. S4, Supplementary Table S9) and the Civil Police (Supplementary Fig. S5, Supplementary Table S9) showed similar age distributions, with most cases concentrated among women aged 18–29 and 30–39, decreasing progressively in older age groups. At the capital level, 2020–2021 SINAN Vitória data showed that 56.5% (42.2–70.8) of physical IPV cases occurred among women aged 18–29 years (Supplementary Fig. S6, Supplementary Table S10), while Civil Police data reported 43.4% (37.7–49.2) in the same age group (Supplementary Fig. S7, Supplementary Table S10). In Pelotas, SINAN data showed a decreasing pattern in physical IPV cases, from 48.3% (30.1–66.5) among women aged 18–29 years to 20.7% (5.9–35.4) among women aged 40–49 years (Supplementary Fig. S8, Supplementary Table S10).

For the age distribution of sexual IPV, 2019 SINAN Brazil showed similar proportions among women aged 18–29 and 30–39 years, followed by progressive decline across older age groups (Fig. 3, Supplementary Table S8). At the state level, SINAN 2019 reported a higher proportion among women aged 30–39 years (40.0% [29.3–50.7]), whereas 2019 Civil Police data indicated that 58.4% (53.8–62.9) sexual IPV cases occurred among women aged 18–29 (Supplementary Figs. S4 and S5, Supplementary Table S9). Sexual IPV cases from SINAN in Vitória and Pelotas were not reported because of insufficient case numbers.

## Discussion

Understanding methodological differences across data sources is critical to improving the measurement of IPV. This study analyzed IPV estimates from different Brazilian data sources, varying in representativeness, questionnaires, and data collection methods. These differences led to discrepant prevalence rates across data sources for both physical and sexual IPV. In terms of age distribution, physical IPV cases were more common among younger women across data sources, while no clear age pattern emerged for sexual IPV cases. Given the methodological heterogeneity across studies, the WHO questionnaire on violence against women^23^ offers a framework for measuring IPV, with standardized definitions and alignments with internationally recommended practices for IPV measurement and participant privacy.

Among the survey data sources, differences in results may partly reflect the lack of standardized data collection instruments, which can influence the measurement of IPV across data sources.^13^ Although some of the studies^30^^,^^33^ used the WHO instrument on violence against women,^23^ others relied on adapted versions,^24^^,^^26^ with variations in the number of items and examples of violent acts presented to respondents. While the full WHO instrument has been validated in Portuguese,^38^ standardizing its shorter, adapted versions remains necessary. The PNS stands out as one of Brazil’s most significant health surveys, with the largest sample size and broad representativeness. It includes all women aged 18 or older, regardless of marital status, at both the national and state levels. However, in terms of estimating IPV, its questionnaire asks only about the perpetrator of the “most severe” episode of violence, which may limit the identification of women who experienced violence in other episodes or involving other perpetrators and may contribute to the lower estimates of physical and sexual IPV. Another methodological factor that could contribute to differences in results is the data collection periods. For instance, LENAD 2006 and 2012 were included as additional sources measuring IPV over the past 12 months, despite being further removed from 2019, the focus year of other studies. The approximately 10% lower prevalence of physical IPV observed in PNS 2019^24^ relative to LENAD 2006^29^ could suggest a decline in physical IPV over this period.^39^ However, this difference cannot be attributed to changes over time alone, as it may also reflect methodological differences between the studies, such as changes in the questionnaires used and whether privacy was guaranteed, which may contribute for discrepancies not only between these two sources but also across other data sources.

The location of interviews and the presence of others during the process are critical factors influencing the quality of IPV data collection. When a partner is present during an interview, the woman may refuse to answer sensitive questions or modify her responses out of fear or under coercion, especially if the partner is a potential aggressor.^5^^,^^40^ Interviews conducted in healthcare facilities or university settings may offer advantages over those conducted at home, as these environments provide greater privacy and a supportive atmosphere for women who disclose experiences of violence.^24^^,^^33^ Also, among the included studies, PNS, Vitória, and LENAD collected data through home-based interviews. Of these, only the Vitória study explicitly reported that interviews were conducted only when women were alone, to ensure privacy during data collection.^30^ Notably, prevalence estimates in Vitória were consistently higher than those observed in the other home-based surveys. However, these studies also differed across several methodological dimensions, which does not allow for the isolation of the effect of interview privacy on prevalence estimates in the present analysis. Future studies specifically designed to examine this methodological aspect could help clarify the extent to which interview setting and privacy conditions influence IPV reporting.

The method of questionnaire administration can also influence IPV prevalence estimates. Studies suggest that telephone interviews often yield higher response rates for sensitive questions than face-to-face interviews, though excluding households without telephones can introduce selection and representativeness bias.^41^ Self-administered questionnaires, compared to interviews, reduce “social desirability” bias, where respondents adjust answers to align with social norms.^42^ This bias is particularly relevant for sensitive topics like violence, which may be stigmatized and underreported in interviews.^6^ While the PNS offers respondents the option of self-administration or interviews for its violence module, further research is needed to evaluate how these methods impact IPV prevalence estimates in Brazil.

The recall period effects were also among the methodological differences across the included data sources. Discrepancies in sexual IPV results for Vitória may stem from differences in recall periods: 24 months (2020–2021, during the COVID-19 pandemic) versus the 12-month recall used by other sources. Although data sources based on lifetime recall were available, they were excluded because they did not align with the study’s focus. In contrast, the Vitoria study was retained because its 24-month recall period was more closely aligned with the 12-month recall used by the other sources.

Regional differences, even within the same country, play a significant role when measuring IPV. In addition to nationally representative surveys, two other surveys were included: one from Vitória (Espírito Santo), a capital city in southeastern Brazil, and another from Pelotas (Rio Grande do Sul), a city in southern Brazil. It is well established that regional differences, sociodemographic and socioeconomic characteristics, and regional public policies influence health outcomes. Therefore, these geographical differences should also be taken into account when interpreting variation in IPV data against women.

Selection criteria and population restrictions across data sources should also be considered when interpreting IPV estimates and their external validity. Some studies included specific population groups, such as married or partnered women, mothers, or populations reached through particular modes of contact. These design choices shape the populations represented in each estimate and should be taken into account when considering the extent to which findings apply to broader populations.

Taken together, these findings indicate that variation in prevalence estimates should be interpreted in the context of multiple methodological characteristics rather than attributed to any single study feature in isolation. Across surveys, differences in target populations, eligibility criteria, measurement instruments, recall periods, interview procedures, and geographic coverage may all influence reported estimates. Similar considerations apply when interpreting differences between survey data and administrative records, although these sources are also shaped by distinct pathways of access, reporting, and case identification.

Differences between survey data and notification records from the Civil Police and SINAN must be understood in context. Administrative systems only include women who sought victim support services, often for severe violence, frequently perpetrated by strangers, which may obscure domestic violence and are subject to underreporting and lack representativeness, especially given the sensitive nature of IPV.^13^ At the national level, a study using survey-based data from PNS 2019 and administrative data from SINAN estimated underreporting in SINAN at 75% for physical violence and 90% for sexual violence.^43^ Discrepancies also exist between Civil Police and Health Sector data. For instance, at the municipal level, a study linking SINAN and Civil Police records in a Brazilian municipality found that the Police reported 8 times as many cases as SINAN during the same period, with fewer than 5% of cases appearing in both databases, indicating that few women sought help from both sectors.^44^ A study on Cybercrime in Nepal also found inconsistencies in reporting and data environments, which highlights how platform-specific (Facebook/Messenger, TikTok, among others) data variability can distort violence estimates across contexts.^45^ Integrating data from Health and Public Security sectors can provide a more comprehensive understanding of IPV cases, revealing different violence patterns and profiles of women, which can inform targeted public policies.^18^^,^^46^^,^^47^

This study initially aimed to analyze IPV underreporting but was limited by significant disparities among data sources. Despite this, investigating these sources remains valuable, as Brazil provides diverse datasets for such analyses. We evaluated surveys at the national, state, capital, and municipal levels, as well as records from police and health systems. A key finding is that differences in survey design and implementation should be considered when interpreting prevalence estimates. Studies using the WHO violence questionnaire incorporated standardized definitions and measurement procedures, providing a methodological framework for future studies.^23^^,^^30^^,^^33^ Previous research often combines administrative data and surveys within the same population to estimate underreporting and evaluate the advantages and limitations of each approach.^7^^,^^48^^,^^49^ Longitudinal studies applying consistent methods over time can also provide insights into changes in reporting patterns.^16^ Administrative and police system data offer insights into societal trends based on health service and police records.^18^ These varied data sources can either support or challenge policy and program development. This study highlights the importance of leveraging these data to inform public policy and improve the measurement of violence.

To strengthen national IPV surveillance, large-scale surveys such as Brazil’s PNS could consider incorporating the full WHO instrument on violence against women, including explicit assessment of different episodes of violence rather than restricting question to the “most severe episode”. Ensuring that interviews are conducted in private settings may also facilitate disclosure of experiences of violence. On a global level, Brazil’s geographic, demographic, and administrative diversity makes it a valuable case study for low- and middle-income countries facing similar challenges in measuring IPV. This heterogeneity highlights the range of methodological and contextual factors that need to be considered in IPV measurement and underscores the importance of developing IPV measurement strategies to diverse contexts worldwide. Policymakers and researchers in other countries can draw on Brazil’s experience to improve data collection methodologies and policy responses. Taken together, these considerations can inform ongoing efforts to strengthen IPV measurement and support evidence-informed prevention and intervention policies both in Brazil and internationally.

This study has several limitations. First, the data sources analyzed differed across multiple methodological dimensions, including target population, minimum age, recall period, historical period, measurement instruments, and interview context. These differences limit direct interpretation of variation in prevalence estimates across sources and do not allow isolation of the effects of specific methodological characteristics, such as questionnaire design, mode of administration, or privacy conditions during data collection. Therefore, the findings should be interpreted as illustrating how multiple methodological differences may influence IPV estimates rather than attributing variation to individual factors. Second, sample sizes and population characteristics varied considerably across data sources. PNS has the largest and most representative sample, while LENAD and Data Senado Research have smaller national samples that may not capture the full population. Additionally, cases of violence reported across data sources are not mutually exclusive, as making them so would further reduce case numbers, particularly for sexual violence. Other specific differences include the 2019 Pelotas study, which exclusively includes mothers in a particular age range, and the LENAD survey, which included only married women. Finally, this study reported data only for women, and a limitation is that it did not include data on race/ethnicity.

Understanding the impact of variability in data collection methods is crucial for improving IPV assessment and shaping effective public policies in Brazil and globally.^50^ Efforts to enhance violence measurement should consider established practices for data collection, consistent survey methodologies and instruments, the use of data from the same geographical level, methodological innovation research, and the implementation of recommendations for future.^51^ Addressing sources of underreporting and strengthening case identification may provide a more comprehensive understanding of violence against women and better reflect the real scope of the issue. This approach can drive meaningful investments in public policies to protect victims, strengthen detection and prevention efforts, and address this significant health risk affecting women worldwide.

### Conclusion

Our findings demonstrate that evaluating methodologically seven data sources from the same country to measure physical and sexual IPV reveals both discrepancies and similarities in prevalence and age distribution of cases. Identifying violence patterns specific to different age groups can help healthcare providers and policymakers design targeted interventions for women experiencing IPV. In the absence of a definitive gold standard for assessing IPV prevalence, it is vital to evaluate the strengths and limitations of the data sources used to estimate IPV prevalence. Repeated assessments using consistent methods over time may also provide additional insights into changes in IPV reporting and prevalence. Further research on measurement methodologies is needed to understand the sources of variation in IPV estimates and better address and correct the underreporting of IPV against women.

## Contributors

CS, NMV, EG, and DCM participated in writing the first draft of the manuscript; CS, NMV, BV, and AT shared primary responsibility for applying analytical methods to produce estimates; CS, NMV, FMCL, BV, MH, and AT, shared primary responsibility for seeking, cataloguing, extracting, or cleaning data; CS, NMV, JM, FMCL, BV, LSF, SIH, EG, DCM, contributed to designing or coding figures and tables; CS, NMV, CVNC, JM, FMCL, BV, LSF, MH, AT, EG, and DCM participated in providing data or critical feedback on data sources; CS, NMV, CVNC, JM, FMCL, BV, LSF, SIH, EG, and DCM participated in providing critical feedback on methods or results; CS, NMV, CVNC, JM, FMCL, BV, LSF, SIH, EG, and DCM contributed to drafting the manuscript or revising it critically for important intellectual content; CS, NMV, SIH, EG, and DCM managing the estimation or publications process. The lead, corresponding, and senior authors had full access to the data in the study and had final responsibility for the decision to submit for publication.

## Data sharing statement

This study used secondary data from publicly available databases. These databases are in the public domain and can be accessed on the internet. More details about the National Health Survey (PNS); the Brazilian National Alcohol and Drugs Survey (LENAD); Data Senado Research (DSR); and the Brazil Information System for Notifiable Diseases (SINAN) could be found at https://ghdx.healthdata.org/.

Civil Police data from Espírito Santo State: access to the data was obtained through a request submitted to the ombudsman’s office for access to data from the State Secretariat for Public Security and Social Defense, based on the Access to Information Law (https://e-ouv.es.gov.br/).

Two data sources were requested through the Proposal Form for Data Access: the 2020–2021 Vitória study and the 2015 Pelotas Birth Cohort, 4-year follow-up.

Code used for these analyses is publicly available online https://github.com/CarolineStein/IPV_in_Brazil_repository.

## Artificial intelligence declaration statement

Generative artificial intelligence (AI) tools, specifically ChatGPT (OpenAI, GPT-5.5), were used as an instrumental aid during manuscript preparation. The tool supported the linguistic and structural revision of the text, contributing to improvements in clarity, organization, and formal presentation. It was not used for the autonomous generation of scientific content, hypothesis development, data interpretation, methodological decision-making, or scientific conclusions. All outputs generated with AI assistance were critically reviewed, verified, and revised by the authors, who retain full responsibility for the accuracy, integrity, and originality of the manuscript.

## Declaration of interests

All authors declare no competing interests.
