# Supplementary material for: Assessing methodological differences in estimating exposure to intimate-partner violence against women in Brazil: a multi-source observational analysis
Source: Lancet Reg Health Am. 2026 Sep 12;63:101632. doi: 10.1016/j.lana.2026.101632 (PMC13589093; doi:10.1016/j.lana.2026.101632)

Supplementary Information for “Assessing methodological differences in estimating exposure to intimate-partner violence against women in Brazil: a multi-source observational analysis"

This appendix provides detailed information on Methods and Results for the publication titled “Assessing methodological differences in estimating exposure to intimate-partner violence against women in Brazil: a multi-source observational analysis”

Table of Contents

Section 1. GATHER and STROBE checklists 3

Table S1. GATHER checklist 3

Table S2. STROBE checklist. 5

Section 2. Intimate partner violence questions used for each data source. 7

Table S3. Physical intimate partner violence questions used for each data source. 7

Table S4. Sexual intimate partner violence questions used for each data source. 8

Section 3. Supplementary information on data sources used in the analysis. 9

1. Overview of each data source 9

2. Detailed data-source-specific procedures for sample weights and confidence interval estimation 10

3. Missingness summary on each data source 13

Section 4. Supplementary tables 15

Table S5. Physical and sexual IPV prevalence by age group, 2020-2021 Vitória study and 2019 Pelotas study. 15

Table S6. Distribution of physical and sexual IPV cases by age group and national survey data sources. 16

Table S7. Distribution of physical and sexual IPV cases by age group, 2020-2021 Vitória study 17

Table S8. Distribution of physical and sexual IPV cases by age group, SINAN 2019 Brazil. 18

Table S9. Distribution of physical and sexual IPV cases by age group and administrative data source, Espirito Santo. 19

Table S10. Distribution of physical and sexual IPV cases by age group and administrative data source. 20

Section 5. Supplementary figures 21

Figure S1. Physical and sexual IPV prevalence by age group, 2020-2021 Vitória. 21

Figure S2. Physical and sexual IPV prevalence by age group, 2019 Pelotas. 22

Figure S3. Distribution of physical and sexual IPV cases by age group, 2020-2021 Vitória study. 23

Figure S4. Distribution of physical and sexual IPV cases by age group, 2019 SINAN Espírito Santo. 24

Figure S5. Distribution of physical and sexual IPV cases by age group, 2019 Civil Police Espírito Santo. 25

Figure S6. Distribution of physical IPV cases by age group, SINAN Vitória. 26

Figure S7. Distribution of physical and sexual IPV cases by age group, 2020-2021 Civil Police Vitória. 27

Figure S8. Distribution of physical IPV cases by age group, 2019 SINAN Pelotas. 28

Section 1. GATHER and STROBE checklists

This study complies with GATHER recommendations, see table S1 below for the GATHER checklist. The GATHER recommendations can be found on the GATHER website.

Table S1. GATHER checklist

Item #	Checklist item	Reported on page #
**Objectives and funding**		
1	Define the indicator(s), populations (including age, sex, and geographic entities), and time period(s) for which estimates were made.	*Methods (Variables and Data sources); Supplementary Information Section 3*
2	List the funding sources for the work.	*Summary*
**Data Inputs**		
For all data inputs from multiple sources that are synthesized as part of the study:		
3	Describe how the data were identified and how the data were accessed.	*Methods (Overview and Data sources); Supplementary Information Section 3*
4	Specify the inclusion and exclusion criteria. Identify all ad-hoc exclusions.	*Methods (Overview and Variables)*
5	Provide information on all included data sources and their main characteristics. For each data source used, report reference information or contact name/institution, population represented, data collection method, year(s) of data collection, sex and age range, diagnostic criteria or measurement method, and sample size, as relevant.	*Methods (Data sources;) Results (Table 1); Supplementary Information Section 2 and 3*
6	Identify and describe any categories of input data that have potentially important biases (e.g., based on characteristics listed in item 5).	*Discussion (Limitations)*
For data inputs that contribute to the analysis but were not synthesized as part of the study:		
7	Describe and give sources for any other data inputs.	*NA*
For all data inputs:		
8	Provide all data inputs in a file format from which data can be efficiently extracted (e.g., a spreadsheet rather than a PDF), including all relevant meta-data listed in item 5. For any data inputs that cannot be shared because of ethical or legal reasons, such as third-party ownership, provide a contact name or the name of the institution that retains the right to the data.	*Data sharing*
**Data analysis**		
9	Provide a conceptual overview of the data analysis method. A diagram may be helpful.	*Methods (Statistical analysis)*
10	Provide a detailed description of all steps of the analysis, including mathematical formulae. This description should cover, as relevant, data cleaning, data pre-processing, data adjustments and weighting of data sources, and mathematical or statistical model(s).	*Methods (Data sources, Statistical analysis)*
11	Describe how candidate models were evaluated and how the final model(s) were selected.	*NA*
12	Provide the results of an evaluation of model performance, if done, as well as the results of any relevant sensitivity analysis.	*NA*
13	Describe methods for calculating uncertainty of the estimates. State which sources of uncertainty were, and were not, accounted for in the uncertainty analysis.	*Methods (Data sources,*

		Statistical analysis), and Supplementary Information Section 3
**14**	State how analytic or statistical source code used to generate estimates can be accessed.	*Data sharing*
**Results and Discussion**		
**15**	Provide published estimates in a file format from which data can be efficiently extracted.	*Data sharing*
**16**	Report a quantitative measure of the uncertainty of the estimates (e.g. uncertainty intervals).	*Results; Supplementary Information Section 4*
**17**	Interpret results in light of existing evidence. If updating a previous set of estimates, describe the reasons for changes in estimates.	*Discussion*
**18**	Discuss limitations of the estimates. Include a discussion of any modelling assumptions or data limitations that affect interpretation of the estimates.	*Discussion*

Table S2. STROBE checklist.

	Item No	Recommendation	Location where item is reported
**Title and abstract**	1	(*a*) Indicate the study's design with a commonly used term in the title or the abstract	Abstract
		(*b*) Provide in the abstract an informative and balanced summary of what was done and what was found	Abstract
**Introduction**			
Background /rationale	2	Explain the scientific background and rationale for the investigation being reported	Introduction
Objectives	3	State specific objectives, including any prespecified hypotheses	Introduction
**Methods**			
Study design	4	Present key elements of study design early in the paper	Overview
Setting	5	Describe the setting, locations, and relevant dates, including periods of recruitment, exposure, follow-up, and data collection	Overview, Survey data source, Administrative data sources, Supplementary Information Section 3
Participants	6	(*a*) Give the eligibility criteria, and the sources and methods of selection of participants	Data sources
Variables	7	Clearly define all outcomes, exposures, predictors, potential confounders, and effect modifiers. Give diagnostic criteria, if applicable	Variables, Supplementary Information Table S3 and S4 and Section 3
Data sources/ measurement	8*	For each variable of interest, give sources of data and details of methods of assessment (measurement). Describe comparability of assessment methods if there is more than one group	Table 1
Bias	9	Describe any efforts to address potential sources of bias	N/A
Study size	10	Explain how the study size was arrived at	N/A
Quantitative variables	11	Explain how quantitative variables were handled in the analyses. If applicable, describe which groupings were chosen and why	Statistical analysis
Statistical methods	12	(*a*) Describe all statistical methods, including those used to control for confounding	Statistical analysis
		(*b*) Describe any methods used to examine subgroups and interactions	N/A
		(*c*) Explain how missing data were addressed	Supplementary Information Section 3
		(*d*) If applicable, describe analytical methods taking account of sampling strategy	Statistical analysis
		(*e*) Describe any sensitivity analyses	N/A
**Results**			
Participants	13*	(a) Report numbers of individuals at each stage of study—eg numbers potentially eligible, examined for eligibility, confirmed eligible, included in the study, completing follow-up, and analysed	Table 1

		(b) Give reasons for non-participation at each stage	N/A
		(c) Consider use of a flow diagram	N/A
Descriptive data	14*	(a) Give characteristics of study participants (eg demographic, clinical, social) and information on exposures and potential confounders	Results
		(b) Indicate number of participants with missing data for each variable of interest	Supplementary Information Section 3
Outcome data	15*	Report numbers of outcome events or summary measures	
Main results	16	(*a*) Give unadjusted estimates and, if applicable, confounder-adjusted estimates and their precision (eg, 95% confidence interval). Make clear which confounders were adjusted for and why they were included	Results
		(*b*) Report category boundaries when continuous variables were categorized	Results
		(*c*) If relevant, consider translating estimates of relative risk into absolute risk for a meaningful time period	N/A
Other analyses	17	Report other analyses done—eg analyses of subgroups and interactions, and sensitivity analyses	N/A
**Discussion**			
Key results	18	Summarise key results with reference to study objectives	Discussion
Limitations	19	Discuss limitations of the study, taking into account sources of potential bias or imprecision. Discuss both direction and magnitude of any potential bias	Discussion
Interpretatio n	20	Give a cautious overall interpretation of results considering objectives, limitations, multiplicity of analyses, results from similar studies, and other relevant evidence	Discussion
Generalizab ility	21	Discuss the generalizability (external validity) of the study results	Discussion
**Other information**			
Funding	22	Give the source of funding and the role of the funders for the present study and, if applicable, for the original study on which the present article is based	Abstract - Funding

Section 2. Intimate partner violence questions used for each data source.

Table S3. Physical intimate partner violence questions used for each data source.

Data source	Question
**Brazil's Information System for Notifiable Diseases (SINAN)**	Physical violence (also referred to as physical abuse, maltreatment, or physical assault): are violent acts, in which physical force is intentionally, not accidentally, used with the aim of injuring, harming, causing pain and suffering, or destroying the person, leaving visible or invisible marks on their body. It can manifest in various forms, such as slaps, pinches, kicks, twists, shoves, throwing objects, strangulations, burns, stabbings, mutilations, among others. Physical violence also includes injuries by firearms (including stray bullet) or by sharp objects. Only spouse, partner, ex-spouse or ex-partner answers were selected as perpetrators.
**National Health Survey (PNS)**	In the past 12 months, did someone:<br>• Slap or smack you?<br>• Push you, grab you forcefully, or throw something at you with the intent to hurt you?<br>• Punch, kick, or drag you by the hair?<br>• Try to or actually strangle, choke, or intentionally burn you?<br>• Threaten or injure you with a knife, firearm, or any other weapon or object?<br>Consider the most severe episode and answer the following questions: Who did this to you? Spouse, partner, ex-spouse or ex-partner answers were selected.
**Data Senado Research**	And in the past 12 months, has any partner or ex-partner:<br>• Hit, pushed, thrown an object, or done anything else to hurt you?<br>• Threatened to use or used a weapon against you?
**Brazilian National Alcohol and Drugs Survey**	In the past 12 months, has your partner ever:<br>• Thrown something at you?<br>• Pushed you, grabbed, or shook you?<br>• Slapped you?<br>• Kicked or bitten you?<br>• Hit or tried to hit you with something?<br>• Burned or scalded you?<br>• Threatened you with a knife or another weapon, including a firearm?<br>• Injured or tried to injure you with a knife or another weapon, including a firearm?
**Civil Police**	Crimes against person: bodily injury: light: against woman – Maria da Penha Law<br>Crimes against the person: bodily injury: against women – Maria da Penha Law<br>Crimes against person: bodily injury: severe: against women – Maria da Penha Law
**Vitória study**	In the past 24 months, has your partner ever:<br>• Slapped you or thrown something at you that could hurt you?<br>• Push you or shoved you?<br>• Did he hurt you with a punch or some object?<br>• Kicked you, dragged you or beat you up?<br>• Choked or burnt you on purpose?<br>• Threatened to use or actually used a firearm, knife or other weapon against you?
**Pelotas study**	In the past 12 months, have your current or former partner ever:<br>• Slapped you or thrown something at you that could hurt you?<br>• Push you or shoved you?<br>• Did he hurt you with a punch or some object?<br>• Kicked you, dragged you or beat you up?<br>• Choked or burnt you on purpose?<br>• Threatened to use or actually used a firearm, knife or other weapon against you?

Table S4. Sexual intimate partner violence questions used for each data source.

Data source	Question
**Brazil's Information System for Notifiable Diseases (SINAN)**	Sexual violence: is any action in which a person, taking advantage of their position of power and using physical force, coercion, intimidation or psychological influence, with or without the use of weapons or drugs, forces another person, of any sex or age, to engage in, witness or participate in any way in sexual interactions, or to use their sexuality in any way for profit, revenge or any other purposes. Sexual violence includes rape, incestuous abuse, sexual harassment, forced marital sex, non-consensual sexual games and erotic practices, child pornography, pedophilia, voyeurism and forced handling, oral, anal or genital penetration, with penis or objects. It also includes coercive or humiliating exposure to lewd acts, exhibitionism, masturbation, erotic language, sexual interactions of any kind, and pornographic material. Sexual violence also covers acts that, through coercion, blackmail, bribery or enticement, prevent the use of any contraceptive method or force marriage, pregnancy, abortion or prostitution; or that limit or annul a person's autonomy and the exercise of their sexual and reproductive rights. Sexual violence is a crime, even when committed by a family member, whether father, mother, stepfather, stepmother, partner or spouse. Only spouse, partner, ex-spouse or ex-partner answers were selected as perpetrators.
**National Health Survey (PNS)**	In the past 12 months, did someone:<br>• Touch, manipulate, kiss, or expose parts of your body against your will?<br>• Threaten or force you to have sexual intercourse or any other sexual acts against your will?<br>Consider the most severe episode and answer the following questions: Who did this to you? Spouse, partner, ex-spouse or ex-partner
**Brazilian National Alcohol and Drugs Survey**	No analyses were performed for sexual IPV due to the very low number of cases identified in the study.
**Data Senado Research**	There were no questions about sexual violence in the past 12 months.
**Civil Police**	Violence perpetrated by anyone, including intimate partner.<br>Crimes against sexual dignity<br>Crimes against sexual dignity: rape<br>Crimes against sexual dignity: rape of a vulnerable<br>Crimes against sexual dignity: sexual harassment<br>Crimes against sexual dignity: attempted rape<br>Crimes against sexual dignity: other crimes against customs<br>Crimes against sexual dignity: obscene act<br>Crimes against sexual dignity: sexual violation through fraud: sexual importunation<br>Crimes against sexual dignity: rape: against women – Maria da Penha Law<br>Crimes against sexual dignity: violent assessment of mode<br>Crimes against sexual dignity: attempted rape: against women – Maria da Penha Law<br>Crimes against sexual dignity: sexual violation through fraud<br>Crimes against sexual dignity: attempt through fraud
**Vitória study**	Intimate partner violence in the past 24 months:<br>• Did your current or former partner physically forced you to have sex when you didn't want to?<br>• Did you have sexual intercourse because you were afraid of what your partner might do?<br>• Did your current or former partner forced you to do something sexual that you found degrading or humiliating?
**Pelotas study**	Intimate partner violence in the past 12 months:<br>• Did your current or former partner physically forced you to have sex when you didn't want to?<br>• Did you have sexual intercourse because you were afraid of what your partner might do?<br>• Did your current or former partner forced you to do something sexual that you found degrading or humiliating?

Section 3. Supplementary information on data sources used in the analysis.

1. Overview of each data source

Survey data sources

The Brazilian National Alcohol and Drugs Survey (LENAD – Levantamento Nacional de Alcool e Drogas, in Portuguese) is a household survey that seeks to detect habits and attitudes regarding the consumption of alcohol and other drugs, also investigating aspects of the physical and mental health of the Brazilian population. To investigate IPV, the survey used an adapted version of the instrument, The Revised Conflict Tactics Scales, and included individuals aged 14 or older who were married or living with a partner. Data were collected in two periods: LENAD I (from November 2005 to April 2006) and LENAD II (2012).

Brazil's National Survey of Health (PNS - Pesquisa Nacional de Saúde, in Portuguese) is the most important household population-based carried out by the Brazilian Institute of Geography and Statistics (IBGE - Instituto Brasileiro de Geografia e Estatística, in Portuguese) with the Brazilian Ministry of Health in 2019. Data collection took place between August 26, 2019, and March 13, 2020. Interviews were carried out in 90,846 households, with a response rate of 96.5% of the initially planned sample. Among those interviewed, 46,869 were women over 18 years of age, who make up the sample used in this study. It used a multistage complex sampling and is representative of Brazil, the Brazilian subunits, and the Federal District.

Data Senado Research was created in 2005 and aims to provide data that helps the Brazilian Senate understand public opinion about issues being discussed. Since 2005, the Institute has carried out a biannual survey about Domestic and Familiar Violence Against Women. In this study, we analyzed data from the Data Senado Survey 2019, which used an adapted version of the WHO instrument on violence against women and carried out 2,400 interviews by phone. The data collection period took place from September 25 to October 4, 2019.

The population-based study in Vitória (369,000 inhabitants), the capital of the state of Espírito Santo, was carried out from January to May 2022 and investigated the violence against women during the COVID-19 pandemic using the WHO instrument on violence against women.

The 2015 Pelotas Birth Cohort is a prospective population-based study of all children born in Pelotas city (325,689 inhabitants) between January 1 and December 31, 2015. In regular follow-up assessments that have been conducted until children were age 6-7 years old, the study monitors participants' health, physical and psychosocial development, and socioeconomic context throughout their lives, starting from the gestational period. This is the fourth study in the Pelotas Birth Cohort program, the largest study of its kind in Latin America.

Administrative data sources

Brazil's Information System for Notifiable Diseases (SINAN - Sistema de Informação de Agravos de Notificação, in Portuguese) is a national information system for reporting and investigating cases of diseases and conditions that are included in the national list of notifiable diseases. Notification is an institutional obligation, and it is the responsibility of services, managers, and/or professionals to carry out compulsory notification in accordance with current legislation. Local management is responsible for defining strategies for monitoring cases and providing support to professionals. All reported cases of interest to the present study occurred in the years 2019, 2020, and 2021.

The Civil Police System from the state of Espírito Santo is part of the State Secretariat for Public Security and Social Defense, a public department whose purpose is to produce knowledge and support public policies for the socioeconomic development of Espírito Santo, a Southeast State with 3,833,712 inhabitants. Access to the data was made through a request via the ombudsman's office for access to data from the State Secretariat for Public Security and Social Defense, based on the Access to Information Law (https://e-ouv.es.gov.br/). All reported cases of interest to the present study occurred in the years 2019, 2020, and 2021.

2. Detailed data-source-specific procedures for sample weights and confidence interval estimation

1st Brazilian National Alcohol and Drugs Survey (LENAD I – Levantamento Nacional de Alcool e Drogas, in Portuguese)

For LENAD 2006, prevalence estimates were calculated using the survey package in R. The sampling weight weight_4, scaled as weight_4/1,000,000, was incorporated into the survey design object, with independent observations specified as ids = ~1. Age-specific prevalence was estimated as the survey-weighted mean of a binary physical IPV indicator using svymean(), and 95% confidence intervals

were obtained with confint(), based on survey-estimated standard errors and Wald-type intervals using the normal approximation.

Separately, the age distribution of IPV cases was calculated among participants classified as physical IPV cases by summing the sampling weights within each age group and dividing by the total weighted number of IPV cases. Confidence intervals were calculated using the standard error for a proportion, followed by p ± 1.96 × SE. These intervals describe the uncertainty around the weighted distribution of cases by age group.

2nd Brazilian National Alcohol and Drugs Survey (LENAD II – Levantamento Nacional de Alcool e Drogas, in Portuguese)

For LENAD 2012, prevalence estimates were calculated using the survey package in R. The sampling weight final_weight, scaled as final_weight/1,000,000, was incorporated into the survey design object, with independent observations specified as ids = ~1. Age-specific prevalence was estimated as the survey-weighted mean of a binary physical IPV indicator using svymean(), and 95% confidence intervals were obtained with confint(), based on survey-estimated standard errors and Wald-type intervals using the normal approximation.

Separately, the age distribution of IPV cases was calculated among participants classified as physical IPV cases by summing the sampling weights within each age group and dividing by the total weighted number of IPV cases. Confidence intervals were calculated using the standard error for a proportion, followed by p ± 1.96 × SE. These intervals describe the uncertainty around the weighted distribution of cases by age group.

Data Senada Research (DSR)

For DSR 2019, age-specific prevalence estimates were calculated using the survey package in R. The sampling weight W2, scaled as W2/1,000,000, was incorporated into the survey design object, with independent observations specified as ids = ~1. Prevalence was estimated as the survey-weighted mean of a binary physical IPV indicator using svymean(), and 95% confidence intervals were obtained using confint(), based on survey-estimated standard errors and Wald-type intervals using the normal approximation.

Separately, the age distribution of IPV cases was calculated among participants classified as physical IPV cases only, by summing the sampling weights within each age group and dividing by the total weighted number of IPV cases. Confidence intervals were calculated using the standard error for a

proportion, followed by $p \pm 1.96 \times SE$. These intervals describe the uncertainty around the weighted distribution of cases by age group.

Brazil's National Survey of Health (PNS - Pesquisa Nacional de Saúde, in Portuguese)

For the 2019 PNS, analyses were performed using Stata's svy commands after declaring the complex survey design with primary sampling units (UPA_PNS), sampling weights (V00291), strata (V0024), and Taylor linearized variance estimation. Age-specific prevalence estimates were obtained from row percentages using svy: tabulate faixaetaria fisica_vpi, row ci and svy: tabulate faixaetaria sexual_vpi, row ci, where the proportion with IPV within each age group represented the prevalence.

The age distribution of cases was estimated using svy: tabulate fisica_vpi faixaetaria, row ci and svy: tabulate sexual_vpi faixaetaria, row ci, where the row corresponding to IPV cases represented the weighted distribution of cases across age groups. For both prevalence and case-distribution estimates, 95% confidence intervals were calculated from survey-adjusted standard errors estimated by Taylor series linearization, accounting for sampling weights, stratification, and clustering.

Vitória study

For the 2020–2021 Vitória study, the 95% CI were calculated based on the formula: CI = prop + [for the upper limit] and - [for the lower limit] × SE. Crude and relative frequencies were used in the descriptive analyses, and the study adopted a multi-stage sampling process. Age distribution of cases was calculated as the number of cases in each age group divided by the total number of cases.

Pelotas study

For the 2019 Pelotas study, prevalence was estimated by age group as the proportion of participants with physical IPV, calculated as the number of cases divided by the age-specific sample size. The 95% confidence intervals were calculated using the standard error for a binomial proportion, $SE = \sqrt{[p(1-p)/n]}$, and applying the normal approximation as $p \pm 1.96 \times SE$. These correspond to unweighted Wald-type confidence intervals. For both physical and sexual IPV, we did not analyze age distribution of cases for the Pelotas study because the sample consists almost entirely of young women, and consequently, nearly all cases occur within this age group.

Brazil's Information System for Notifiable Diseases (SINAN - Sistema de Informação de Agravos de Notificação, in Portuguese)

Age distribution of cases was calculated as the number of cases in each age group divided by the total number of cases. Confidence intervals were calculated using $SE = \sqrt{[p(1-p)/N]}$ and $p \pm 1.96 \times SE$, corresponding to crude Wald-type intervals based on the normal approximation.

Civil Police System

Age distribution of cases was calculated as the number of cases in each age group divided by the total number of cases. Confidence intervals were calculated manually using $SE = \sqrt{[p(1-p)/N]}$ and $p \pm 1.96 \times SE$, corresponding to crude Wald-type intervals based on the normal approximation.

3. Missingness summary on each data source

Brazilian National Alcohol and Drugs Survey (LENAD), Data Senado Research (DSR), and Brazil's National Survey of Health (PNS)

For all national surveys data sources, publicly available individual-level microdata were accessed and used for the analyses. The released dataset did not contain missing data for the variables included in this study; therefore, no additional procedures for handling missing data were required.

Vitória study

For the 2020–2021 Vitória study, estimates of physical and sexual IPV by age group for all 1,086 women were included, non-missing data were used for each variable. No imputation method was used.

Pelotas study

For the 2015 Pelotas Birth Cohort Study, IPV prevalence estimates were based on available valid IPV outcome data at the 48-month follow-up. Participants without valid IPV information were excluded from the corresponding prevalence analyses, and no imputation method was reported.

Brazil's Information System for Notifiable Diseases (SINAN)

For the SINAN data source, all available records containing the required information on type of violence and age group were included in the analyses. Estimates were calculated using the complete set of non-missing observations and analytic category. No imputation or other missing-data replacement procedure was applied.

Civil Police System

For the Civil Police data source, all available records containing the required information on type of violence and age group were included in the analyses. Estimates were calculated using the complete set

of non-missing observations and analytic category. No imputation or other missing-data replacement procedure was applied.

Section 4. Supplementary tables

Table S5. Physical and sexual IPV prevalence by age group, 2020-2021 Vitória study and 2019 Pelotas study.

Data source	Age group	Study sample	Cases	Prevalence (95% CI)
**Physical IPV**<br>**2020 – 2021 Vitória study**	18 – 29	202	27	13·4 (9·3 – 18·8)
	30 – 39	204	21	10·3 (6·8 – 15·3)
	40 – 49	222	17	7·7 (4·8 – 12·0)
	50 – 59	196	19	9·7 (6·3 – 14·7)
	60 – 99	262	14	5·3 (3·2 – 8·8)
**Sexual IPV**<br>**2020 – 2021 Vitória study**	18 – 29	202	18	8·9 (5·7 – 13·7)
	30 – 39	204	13	6·4 (3·7 – 10·7)
	40 – 49	222	18	8·1 (5·2 – 12·5)
	50 – 59	196	13	6·6 (3·9 – 11·1)
	60 – 99	262	9	3·4 (1·8 – 6·5)
**Physical IPV**<br>**2019 Pelotas study**	18 – 29	1821	137	7·5 (6·3 – 8·7)
	30 – 39	1963	119	6·1 (5·0 – 7·1)
	40 – 49	481	25	5·2 (3·2 – 7·2)
**Sexual IPV**<br>**2019 Pelotas study**	18 – 29	1821	23	1·3 (0·8 – 1·8)
	30 – 39	1963	27	1·4 (0·9 – 1·9)
	40 – 49	481	8	1·7 (0·5 – 2·8)

Table S6. Distribution of physical and sexual IPV cases by age group and national survey data sources.

Data source	Age group	Cases	Expanded cases	Total cases	Expanded total cases	Case distribution
**2006 LENAD**	16 – 29	31	1066935	77	3088726	34·5 (34·5 – 34·6)
	30 – 39	25	1056876	77	3088726	34·2 (34·2 – 34·3)
	40 – 49	12	526578	77	3088726	17·0 (17·0 – 17·1)
	50 – 59	5	233791	77	3088726	7·6 (7·5 – 7·6)
	60 – 99	4	204547	77	3088726	6·6 (6·6 – 6·7)
**2012 LENAD**	16 – 29	33	898740	78	2702680	33·3 (33·2 – 33·3)
	30 – 39	22	809955	78	2702680	30·0 (29·9 – 30·0)
	40 – 49	9	397857	78	2702680	14·7 (14·7 – 14·8)
	50 – 59	11	432491	78	2702680	16·0 (16·0 – 16·0)
	60 – 99	3	163637	78	2702680	6·1 (6·0 – 6·1)
**2019 DSR**	16 – 29	64	2319553	184	6185926	37·5 (37·5 – 37·5)
	30 – 39	55	1575698	184	6185926	25·5 (25·4 – 25·5)
	40 – 49	35	866886	184	6185926	14·0 (14·0 – 14·0)
	50 – 59	16	678669	184	6185926	11·0 (10·9 – 11·0)
	60 – 99	14	745121	184	6185926	12·0 (12·0 – 12·1)
**Physical IPV 2019 PNS**	18 – 29	327	648426	1000	1876736	34·5 (28·4 – 41·2)
	30 – 39	322	604415	1000	1876736	32·2 (27·3 – 37·6)
	40 – 49	197	404277	1000	1876736	21·5 (17·1 – 26·8)
	50 – 59	107	136880	1000	1876736	7·3 (5·2 – 10·1)
	60 – 99	47	82740	1000	1876736	4·4 (2·8 – 6·8)
**Sexual IPV 2019 PNS**	18 – 29	64	144473	244	471490	30·6 (18·9 – 45·6)
	30 – 39	70	93347	244	471490	19·8 (12·2 – 30·5)
	40 – 49	57	156269	244	471490	33·1 (16·5 – 55·5)
	50 – 59	36	47795	244	471490	10·1 (5·8 – 17·3)
	60 – 99	17	29605	244	471490	6·3 (2·5 – 14·9)

Table S7. Distribution of physical and sexual IPV cases by age group, 2020-2021 Vitória study.

Data source	Age group	Cases	Total cases	Case distribution
**Physical IPV 2020 – 2021 Vitória study**	18 – 29	27	98	27·6 (19·6 – 37·2)
	30 – 39	21	98	21·4 (14·4 – 30·7)
	40 – 49	17	98	17·3 (11·1 – 26·2)
	50 – 59	19	98	19·4 (12·7 – 28·4)
	60 – 99	14	98	14·3 (8·6 – 22·7)
**Sexual IPV 2020 – 2021 Vitória study**	18 – 29	18	71	25·4 (16·6 – 36·7)
	30 – 39	13	71	18·3 (10·9 – 29·0)
	40 – 49	18	71	25·4 (16·6 – 36·7)
	50 – 59	13	71	18·3 (10·9 – 29·0)
	60 – 99	9	71	12·7 (6·7 – 22·6)

Table S8. Distribution of physical and sexual IPV cases by age group, SINAN 2019 Brazil.

Data source	Age group	Cases	Total cases	Case distribution
**Physical IPV 2019 SINAN Brazil**	16 – 29	26776	62265	43·0 (42·6 – 43·4)
	30 – 39	19844	62265	31·9 (31·5 – 32·2)
	40 – 49	10572	62265	17·0 (16·7 – 17·3)
	50 – 59	3615	62265	5·8 (5·6 – 6·0)
	60 – 99	1458	62265	2·3 (2·2 – 2·5)
**Sexual IPV 2019 SINAN Brazil**	18 – 29	1047	3141	33·3 (31·7 – 35·0)
	30 – 39	1026	3141	32·7 (31·0 – 34·3)
	40 – 49	681	3141	21·7 (20·2 – 23·1)
	50 – 59	268	3141	8·5 (7·6 – 9·5)
	60 – 99	119	3141	3·8 (3·1 – 4·5)

Table S9. Distribution of physical and sexual IPV cases by age group and administrative data source, Espirito Santo.

Data source	Age group	Cases	Total cases	Case distribution
**Physical IPV 2019 SINAN Espírito Santo**	18 – 29	470	1311	35·9 (33·3 – 38·4)
	30 – 39	462	1311	35·2 (32·7 – 37·8)
	40 – 49	235	1311	17·9 (15·8 – 20·0)
	50 – 59	106	1311	8·1 (6·6 – 9·6)
	60 – 99	38	1311	2·9 (2·0 – 3·8)
**Sexual IPV 2019 SINAN Espírito Santo**	18 – 29	20	80	25·0 (15·5 – 34·5)
	30 – 39	32	80	40·0 (29·3 – 50·7)
	40 – 49	14	80	17·5 (9·2 – 25·8)
	50 – 59	9	80	11·2 (4·3 – 18·2)
	60 – 99	5	80	6·2 (·9 – 11·6)
**Physical IPV 2019 Civil Police Espírito Santo**	18 – 29	469	1106	42·4 (39·5 – 45·3)
	30 – 39	332	1106	30·0 (27·3 – 32·7)
	40 – 49	181	1106	16·4 (14·2 – 18·5)
	50 – 59	82	1106	7·4 (5·9 – 9·0)
	60 – 99	42	1106	3·8 (2·7 – 4·9)
**Sexual IPV 2019 Civil Police Espírito Santo**	18 – 29	262	449	58·4 (53·8 – 62·9)
	30 – 39	93	449	20·7 (17·0 – 24·5)
	40 – 49	59	449	13·1 (10·0 – 16·3)
	50 – 59	20	449	4·5 (2·5 – 6·4)
	60 – 99	15	449	3·3 (1·7 – 5·0)

Table S10. Distribution of physical and sexual IPV cases by age group and administrative data source.

Data source	Age group	Cases	Total cases	Case distribution
**Physical IPV**<br>**2020 – 2021 SINAN Vitória**	18 – 29	26	46	56·5 (42·2 – 70·8)
	30 – 39	10	46	21·7 (9·8 – 33·7)
	40 – 49	6	46	13·0 (3·3 – 22·8)
	50 – 59	3	46	6·5 (0·0 – 13·7)
	60 – 99	1	46	2·2 (0·0 – 6·4)
**Physical IPV**<br>**2020 – 2021 Civil Police Vitória**	18 – 29	126	290	43·4 (37·7 – 49·2)
	30 – 39	91	290	31·4 (26·0 – 36·7)
	40 – 49	47	290	16·2 (12·0 – 20·4)
	50 – 59	19	290	6·6 (3·7 – 9·4)
	60 – 99	7	290	2·4 (0·6 – 4·2)
**Sexual IPV**<br>**2020 – 2021 Civil Police Vitória**	18 – 29	99	153	64·7 (57·1 – 72·3)
	30 – 39	32	153	20·9 (14·5 – 27·4)
	40 – 49	17	153	11·1 (6·1 – 16·1)
	50 – 59	2	153	1·3 (0·0 – 3·1)
	60 – 99	3	153	2·0 (0·0 – 4·2)
**Physical IPV**<br>**2019 SINAN Pelotas**	18 – 29	14	29	48·3 (30·1 – 66·5)
	30 – 39	9	29	31·0 (14·2 – 47·9)
	40 – 49	6	29	20·7 (5·9 – 35·4)

Section 5. Supplementary figures

Figure S1. Physical and sexual IPV prevalence by age group, 2020-2021 Vitória.

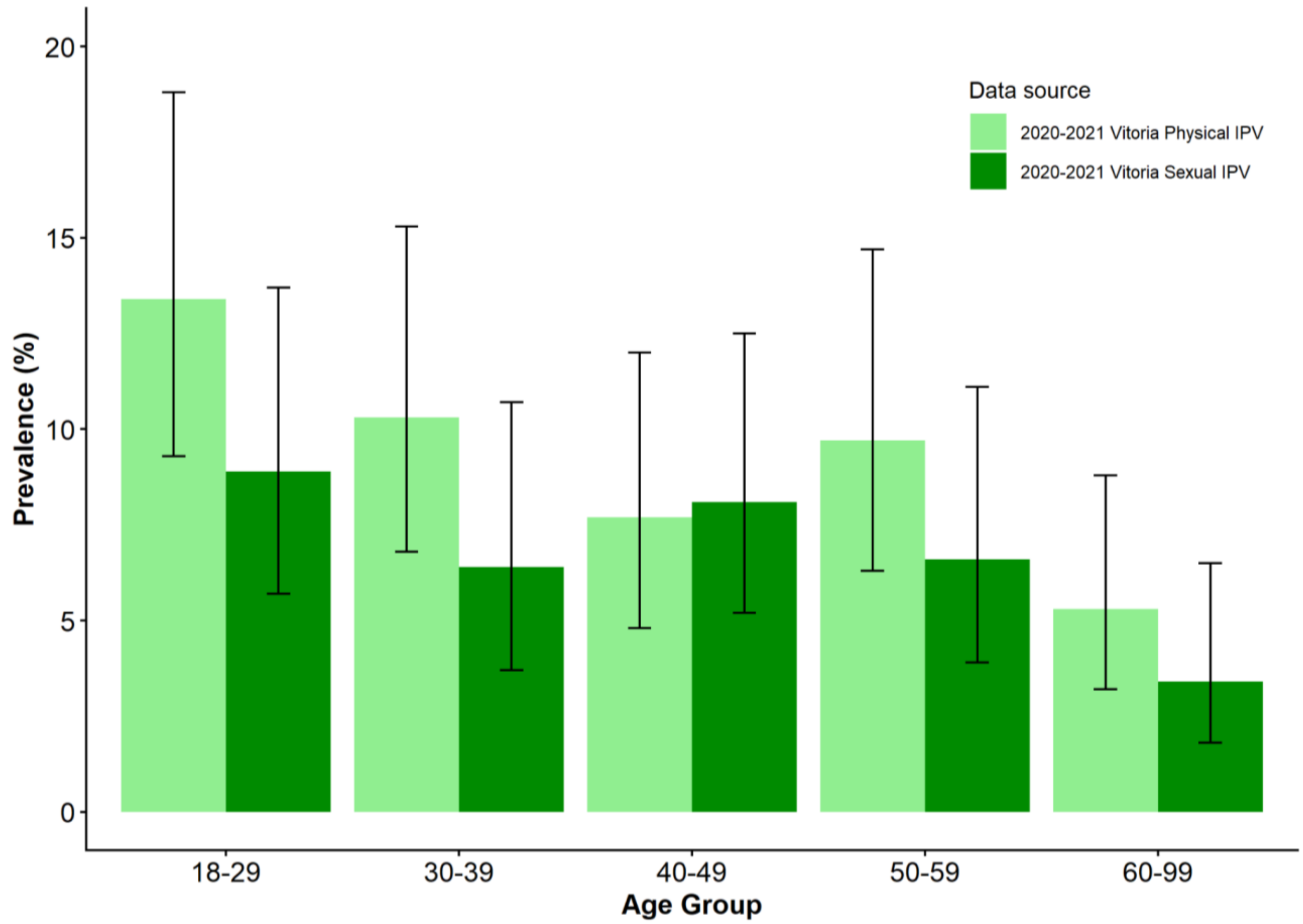

Figure S2. Physical and sexual IPV prevalence by age group, 2019 Pelotas.

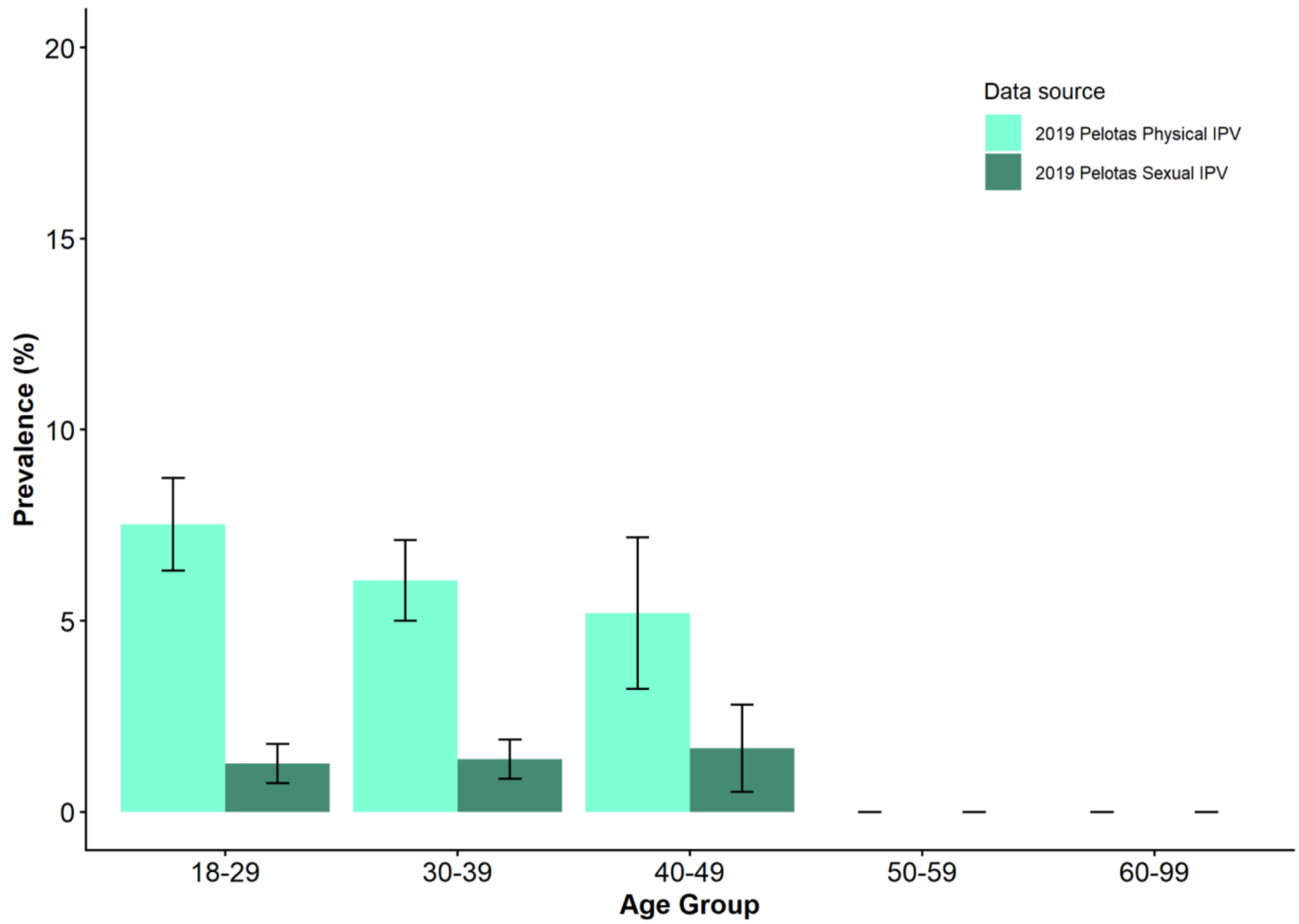

Figure S3. Distribution of physical and sexual IPV cases by age group, 2020-2021 Vitória study.

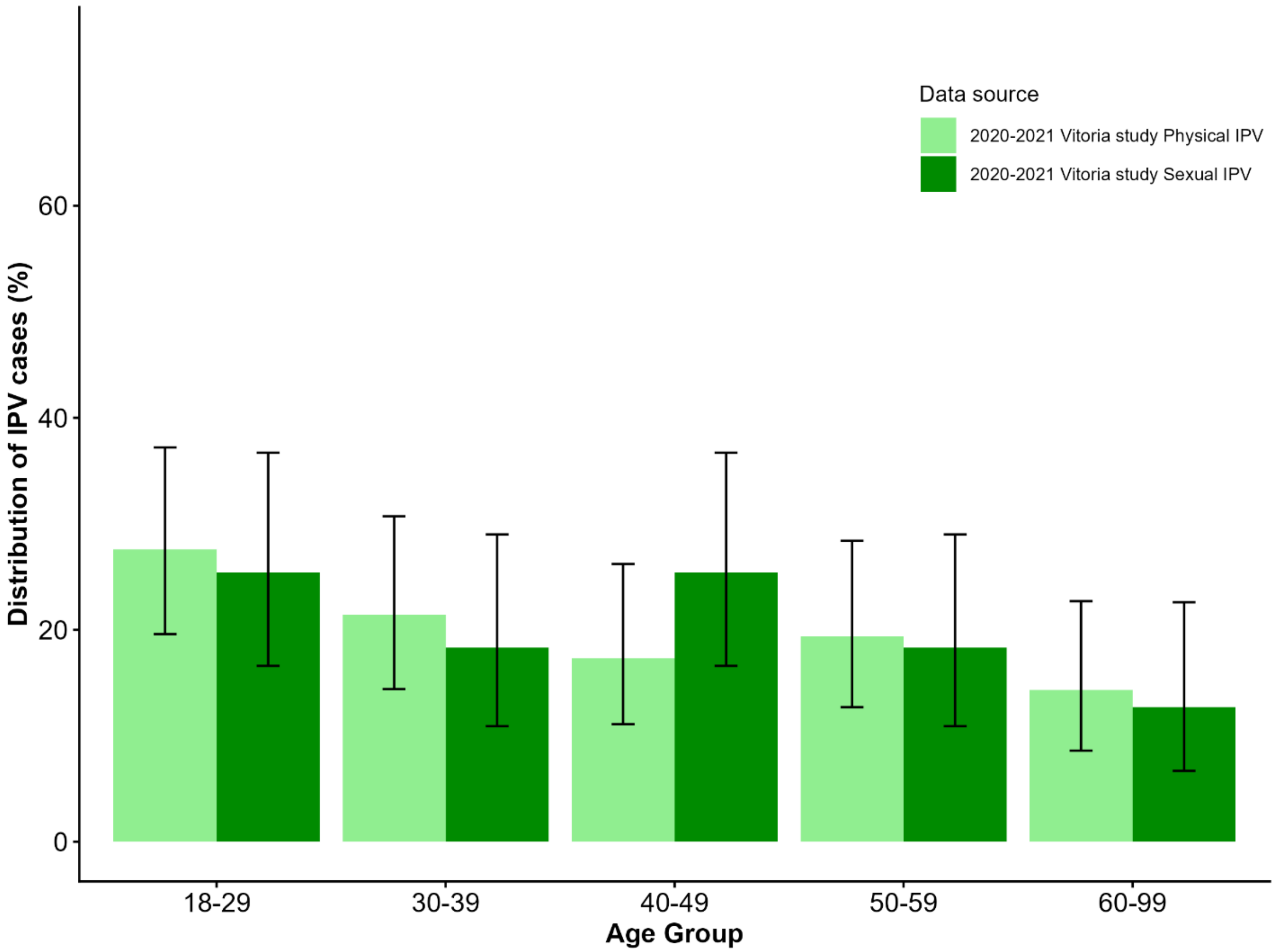

Figure S4. Distribution of physical and sexual IPV cases by age group, 2019 SINAN Espírito Santo.

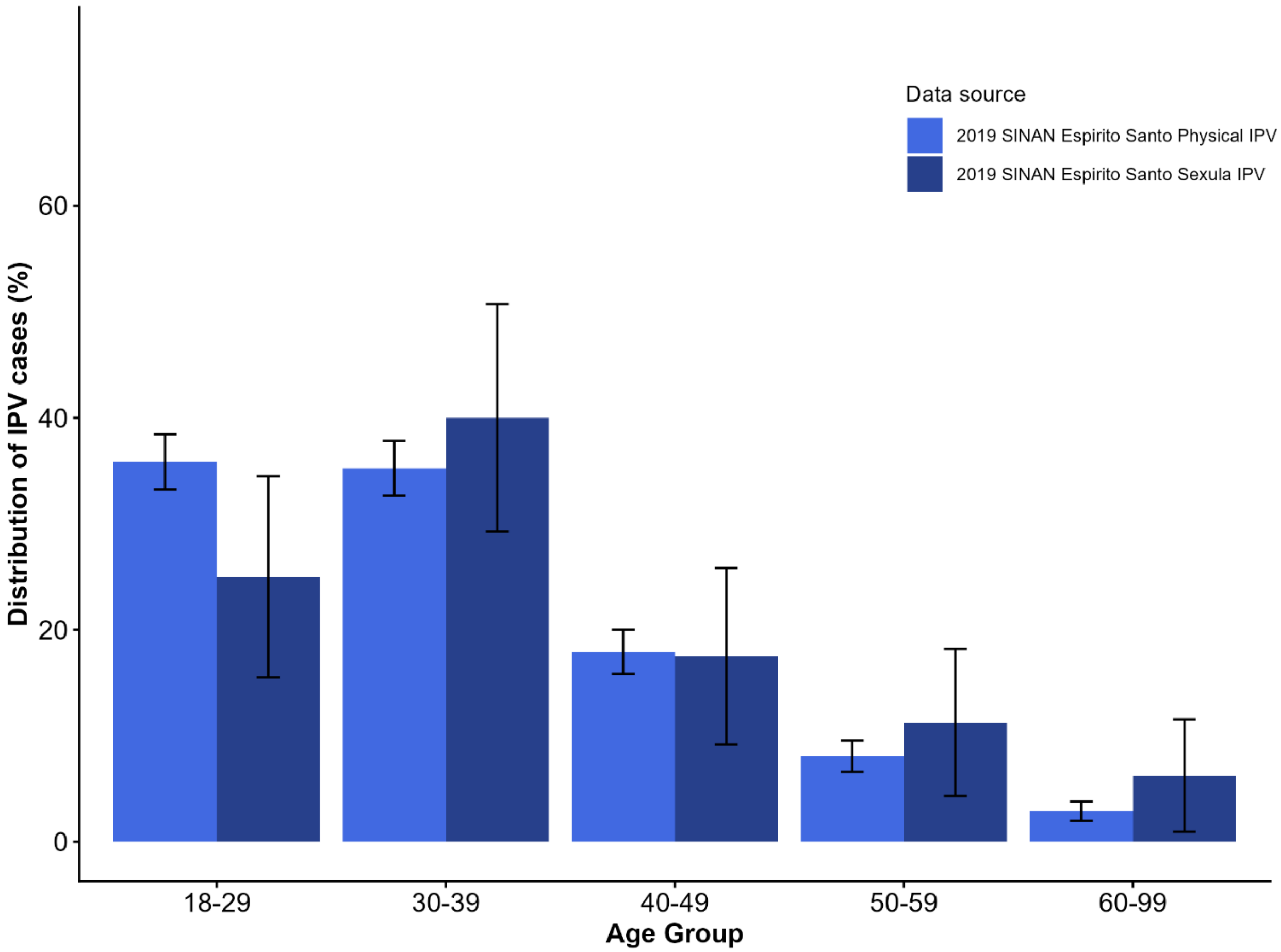

Figure S5. Distribution of physical and sexual IPV cases by age group, 2019 Civil Police Espírito Santo.

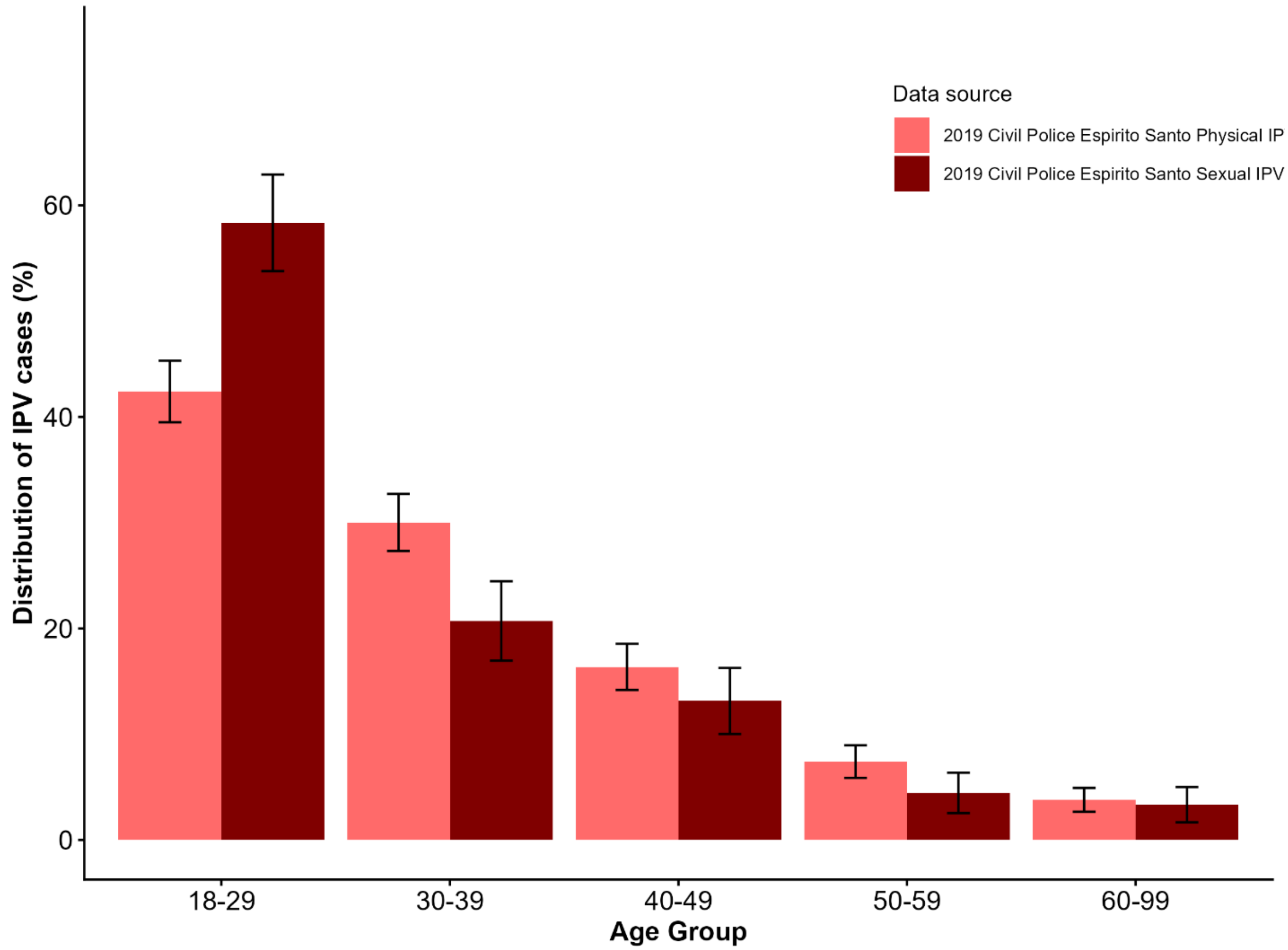

Figure S6. Distribution of physical IPV cases by age group, SINAN Vitória.

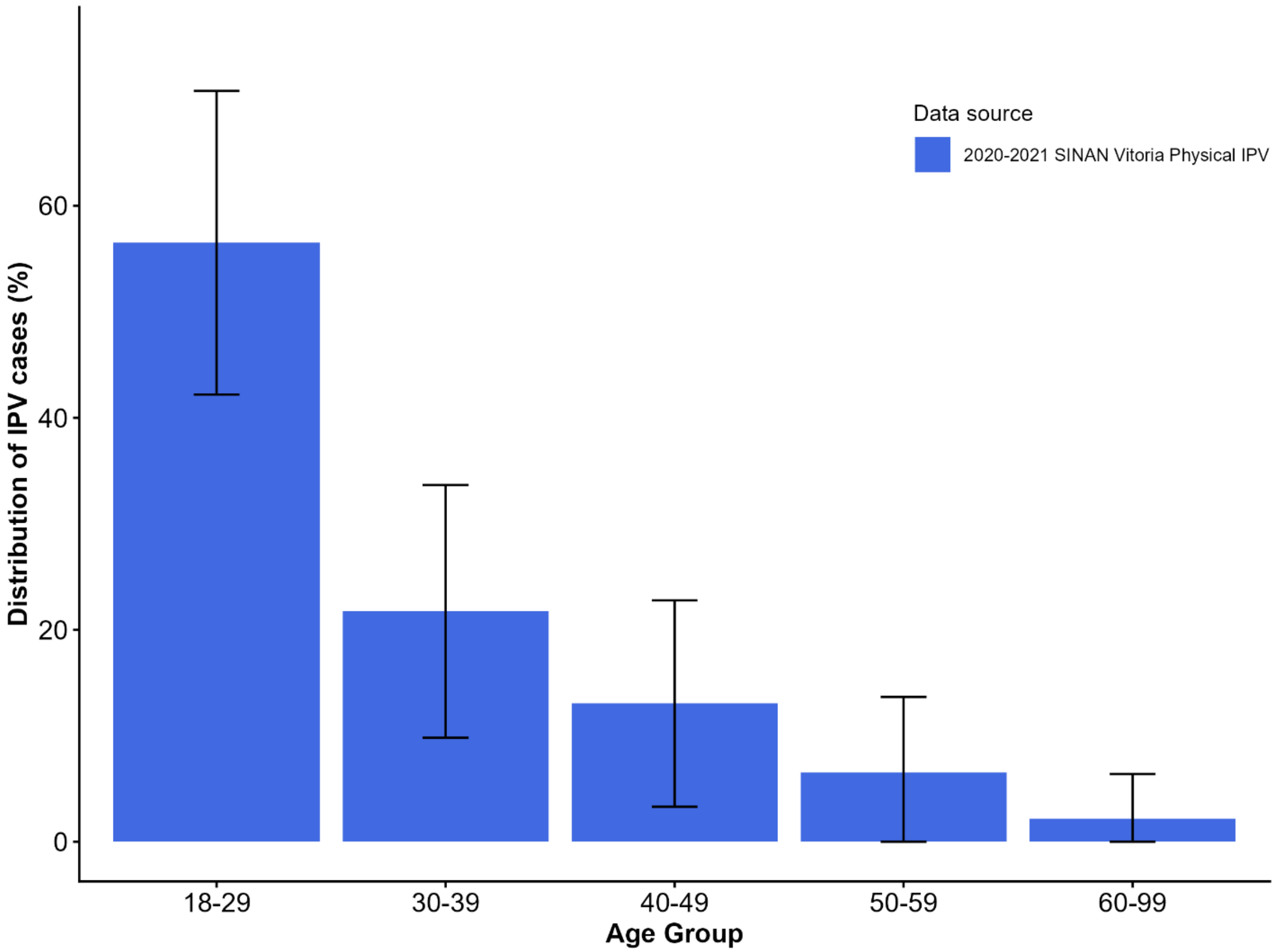

Figure S7. Distribution of physical and sexual IPV cases by age group, 2020-2021 Civil Police Vitória.

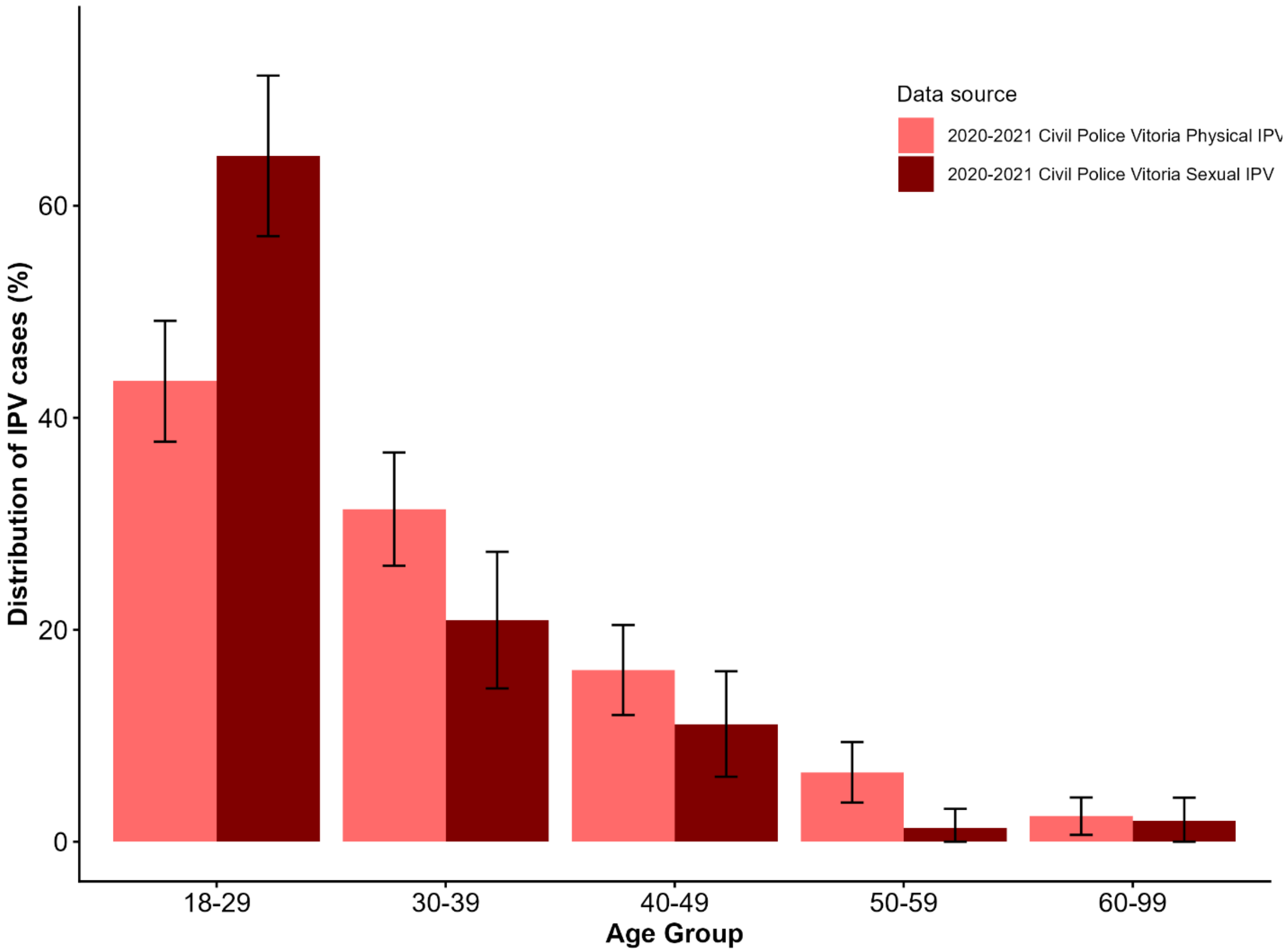

Figure S8. Distribution of physical IPV cases by age group, 2019 SINAN Pelotas.

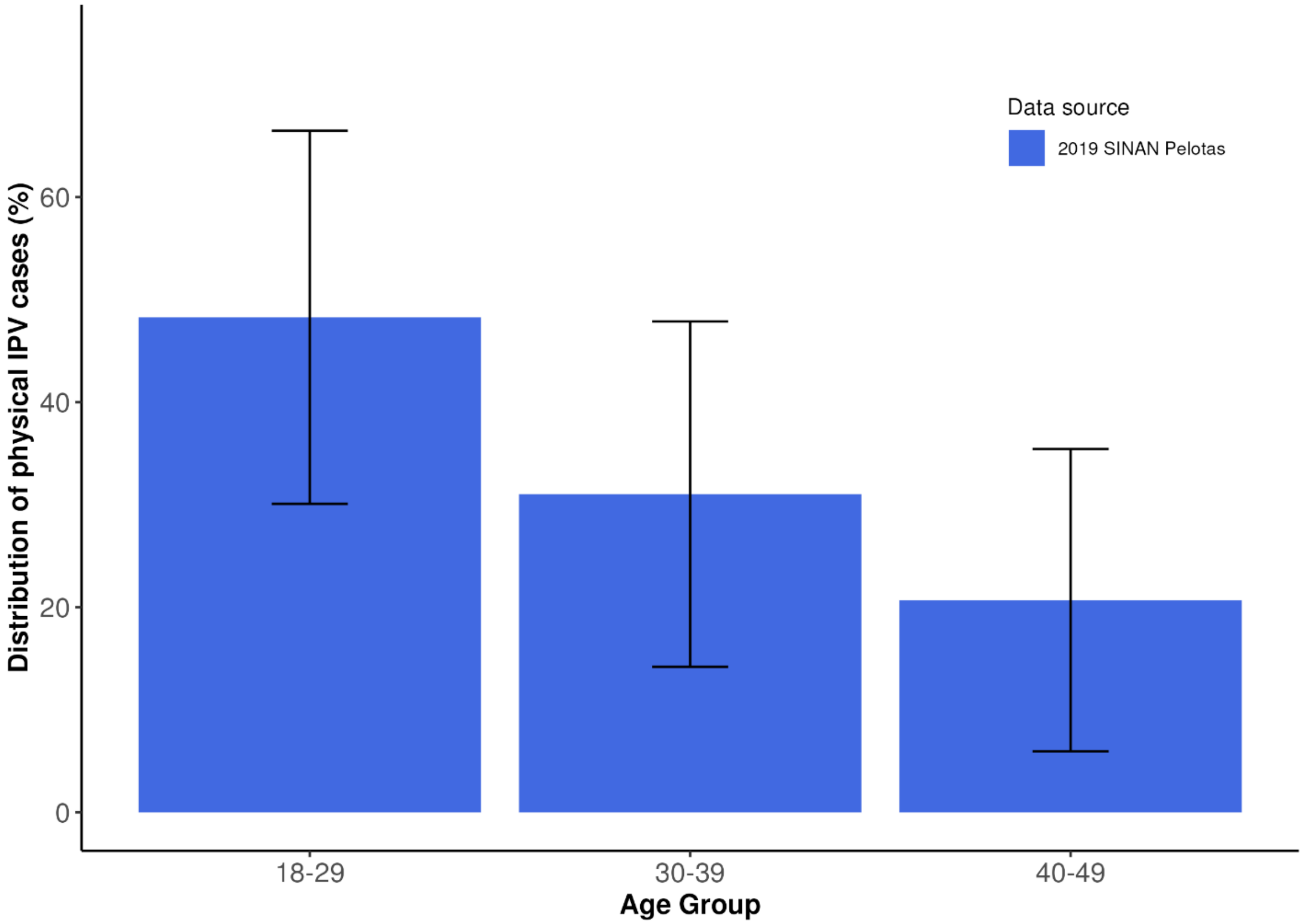

Supplement: Supplementary file 1 [file mmc1.pdf]
